# Chronic Stress-Induced Depression and Anxiety Priming Modulated by Gut-Brain-Axis Immunity

**DOI:** 10.3389/fimmu.2021.670500

**Published:** 2021-06-24

**Authors:** Susan Westfall, Francesca Caracci, Molly Estill, Tal Frolinger, Li Shen, Giulio M. Pasinetti

**Affiliations:** ^1^ Department of Neurology, Icahn School of Medicine at Mount Sinai, New York, NY, United States; ^2^ Department of Neuroscience, Icahn School of Medicine at Mount Sinai, New York, NY, United States; ^3^ Geriatric Research, Education and Clinical Center, James J. Peters Veterans Affairs Medical Center, Bronx, NY, United States

**Keywords:** probiotic, nutraceutical, innate lymphocyte cells, psychiatry, microbiota

## Abstract

Chronic stress manifests as depressive- and anxiety-like behavior while recurrent stress elicits disproportionate behavioral impairments linked to stress-induced immunological priming. The gut-brain-microbiota-axis is a promising therapeutic target for stress-induced behavioral impairments as it simultaneously modulates peripheral and brain immunological landscapes. In this study, a combination of probiotics and prebiotics, known as a synbiotic, promoted behavioral resilience to chronic and recurrent stress by normalizing gut microbiota populations and promoting regulatory T cell (Treg) expansion through modulation of ileal innate lymphoid cell (ILC)3 activity, an impact reflecting behavioral responses better than limbic brain region neuroinflammation. Supporting this conclusion, a multivariate machine learning model correlatively predicted a cross-tissue immunological signature of stress-induced behavioral impairment where the ileal Treg/T helper17 cell ratio associated to hippocampal chemotactic chemokine and prefrontal cortex IL-1β production in the context of stress-induced behavioral deficits. In conclusion, stress-induced behavioral impairments depend on the gut-brain-microbiota-axis and through ileal immune regulation, synbiotics attenuate the associated depressive- and anxiety-like behavior.

## Introduction

Chronic stress imposes persistent immunological changes to the periphery and brain priming the host to disproportionally respond to recurrent subthreshold stresses (1). The most common psychiatric responses to chronic stress include mood disorders such as major depressive disorder (MDD) and anxiety, while future sensitivity to recurring stressors can manifest as post-traumatic stress disorder (PTSD). Gut microbiota modifying agents including probiotics and prebiotics attenuate symptoms of stress-induced behavioral impairment (2, 3), yet the mechanisms remain in their infancy. Depression and anxiety associated with chronic stress have complex etiologies linked to maladaptive responses in neurovascular architecture, neuroendocrine signaling, and immune signaling (4, 5). Chronic stress has been causally linked to inflammation in the periphery (6, 7) and the brain (8) and this chronic low-grade inflammation resulting from chronic stress has become a major risk factor for the evolution of stress-induced psychiatric impairment (9). Inflammation could act as a therapeutic target for depression and anxiety; however, the unique disparate immune regulation of the periphery and the brain provides pharmacological challenges (10). Notably, the microbiome-gut-brain-axis can indiscriminately regulate maladaptive immune responses in both the periphery and the brain (11) making it an attractive therapeutic target. This multifaceted strategy could reform the reductionist approaches of current therapeutic regimes for psychiatric impairment, which classically target only one protein or receptor leading to inconsistent and poor clinical outcomes (12).

Chronic stress priming to recurrent psychological impairment has been attributed to microglia activation (13, 14), associated to sterile inflammation in the hippocampus (15), peripheral IL-6 production (6), activation of major histocompatibility complex (MHC)II^+^ CD11c^+^ dendritic cells and Ly6C^hi^ monocytes (10), proinflammatory leukocytes (16), a migratory phenotype in myeloid cells (17) and trafficking of myeloid cells from the spleen into limbic brain regions (18). Expansion of circulating inflammatory monocytes may also confer susceptibility to recurrent stressful episodes (19) while infiltration of chemokine receptor 2 (CCR2^+^) Ly6C^hi^ monocytes into the brain and their subsequent differentiation to IL-1β producing macrophages may exasperate neuroinflammatory phenotypes to recurrent challenges (20). It has also been proposed that the adaptive immune system may store a stressor’s immunological memory facilitating protection against or sensitivity to future stress exposures (21).

The composition of the gut microbiota is an important influence for managing psychiatric health (22, 23). Anxiety-like behavior can be transferred by the gut microbiota (24) and attenuated by supplementation with certain probiotics (i.e. psychobiotics) (25–27). In general, reconstitution of germ-free mice with commensal microbiota or treatment with psychobiotics promotes IL-10 production and expansion of Tregs (27, 28). Communication between the gut microbiota and the immune system occurs through both cognate interactions and *via* its secreted metabolome including tryptophan metabolites, polyphenolic metabolites and short chain fatty acid production, which can modulate immune cells’ responsiveness (29). Tryptophan catabolism is controlled by the gut microbiota producing metabolites that interact with the aryl hydrocarbon receptor (AHR). These interactions transcriptionally regulate innate immune cells and intestinal epithelial cells influencing the balance of pro- and anti-inflammatory cell types in the gastrointestinal associated lymphoid tissue (GALT) (30). We also identified that synbiotic-specific polyphenolic metabolites alter the Th17/Treg ratio, possibly through mechanisms involving the AHR (28). Although many pathways have been identified, there remains an innate complexity to the relationship between microbial metabolites and the immune system. Likewise, direct interactions have been identified between microbial metabolites and innate lymphoid cells (ILCs), dendritic cells, macrophages, monocytes, neutrophils and naïve lymphocytes [reviewed in (29)] diluting the discovery of direct gut microbiome-gut-brain axis interactions in the context of chronic or recurrent stress.

In a previous study, we showed that a synbiotic composed of *Lactobacillus fermentum, Bifidobacterium longum* and a polyphenol-rich prebiotic attenuated behavioral deficits to chronic stress by altering the Treg/Th17 ratio in the ileum through mechanisms potentially implicating the AHR (28). The prebiotic used was a Botanical Derived Polyphenolic Preparation (BDPP) composed of grape seed extract, concord grape juice and resveratrol, a cocktail we previously shown to be neuroprotective (31, 32). Our previous studies also showed that the plasma and brain bioavailability of BDPP’s bioactive polyphenolic metabolites is greatly enhanced when administered in conjunction with probiotics as a synbiotic (28). In the current study, correlative gut-brain-axis associations in the context of recurrent stress-induced anxiety and depression were formulated using an unbiased machine learning algorithm. This technique was employed to build generalized associations between chronic and recurrent stress, gut microbiota composition and inflammatory markers in a manner that considers the variation between individuals within groups and the synergistic activities between the periphery and brain. This analysis created a set of putative mechanisms to be addressed in future work demonstrating that the gut-brain-axis cannot be considered as isolated tissues, but a synergistic system of gut, periphery and brain that can ultimately change the behavioral signature of stress-induced depression and anxiety.

## Materials and Methods

### Bacteria and Fermentation Conditions

The bacterial cell lines *Lactobacillus plantarum* ATCC 793 (Lp793) and *Bifidobacterium longum* ATCC 15707 (Bl15707) were cultivated from frozen stock in Man-Rogosa-Sharpe (MRS) media and MRS with 0.05% cysteine, respectively, in an anaerobic incubator at 37°C as previously described (28).

### Animal Husbandry

C57BL/6J male mice were purchased from The Jackson Laboratory (age 8 weeks; Bar Harbor, ME, USA) and group housed in the centralized animal care facility of the Center for Comparative Medicine and Surgery at the Icahn School of Medicine at Mount Sinai. Male mice were exclusively used in this study to normalize the behavioral effects due to stress; further studies will be conducted to directly compare sex in stress-induced behavioral deficits. All animals were maintained on a 12-h light/dark cycle in a temperature-controlled (20 ± 2°C) vivarium with access to food and water *ad libitum.* All procedures, protocols and behavioral experiments were approved by the Mount Sinai Institutional Animal Care and Use Committee (IACUC).

Animals were fed a polyphenol-free diet for the duration of all experiments (**Table S1**). Following the 2-week stabilization and acclimatization period for the mice, animals were placed on their respective treatment for 2 weeks prior to starting the stress protocol. The Bioactive Dietary Polyphenol Preparation (BDPP) was comprised of 1% *w*/*v* grape seed polyphenol extract (GSPE; Healthy Origins), 1% *w*/*v* resveratrol (BulkSupplements.com) and a 5% *w*/*v* concord grape extract (AA Pharmachem, San Diego, USA) made in sterile water. All tested compounds were analyzed by liquid chromatography–mass spectrometry and archived as previously reported (28, 31) in compliance with National Institutes of Health, National Center for Complementary and Integrative Health (Bethesda, MD, USA) product integrity guidelines. Probiotic doses for mice were prepared by growing cultures in bulk as previously described (28). The bacteria were incorporated into the animals’ drinking water at a final dosage of 1.0x10 (9) CFU/day per bacterium, calculated based on the average daily water consumption per cage, which was recalculated and replaced daily. The synbiotic was composed of BDPP and the probiotics and was replaced daily. Bacteria cultures were prepared in bulk, aliquoted at a known density and frozen at -80°C until needed. This viability of this preparation was tested by freezing bacterial stocks as indicated and replating from thawed samples and preforming colony counts.

### Chronic Unpredictable Stress Protocol

**Figure 1:**
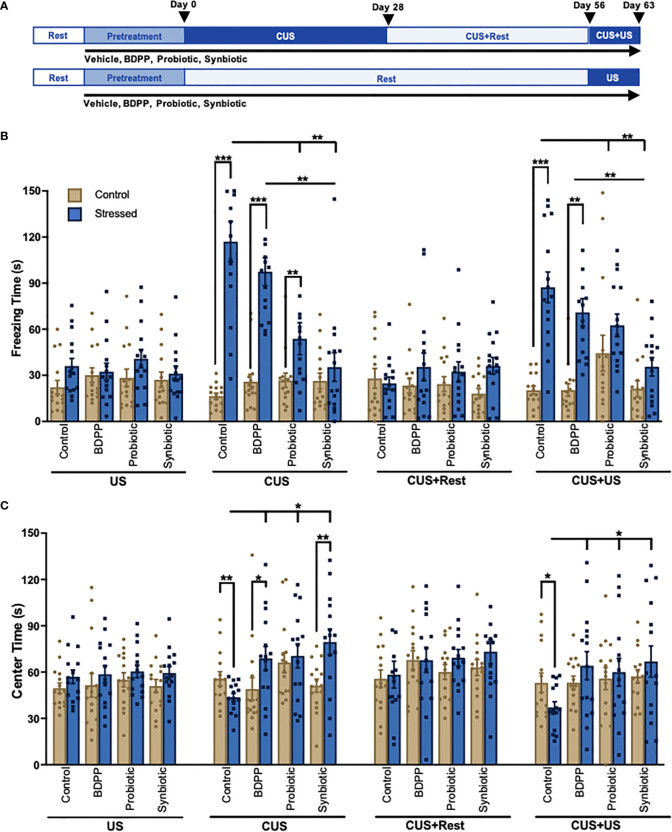
A Synbiotic Attenuates Chronic-Stress Induced Psychological Deficits. **(A)** Chronic and recurrent stress are modelled using the chronic unpredictable stress (CUS) protocol. Following 2 weeks of rest and 2 weeks of pretreatment with the BDPP, probiotics or synbiotics, mice are exposed to 28 days of random mild unpredictable stressors (CUS) following by 28 days of rest (CUS+Rest) and a re-stimulation of 7 days of unpredictable stress (US) modelling recurrent stress (CUS+US). The final CUS+US timepoint can be compared to the 7-day subthreshold US. Each group contained *n* = 16 animals. Behavior phenotypes were assessed using the **(B)** forced swim test for depressive-like behavior and **(C)** open field test for anxiety-like behavior where for each group there are *n* = 16 mice +/- SEM and significance is determined with a two-way ANOVA and Tukey’s post-hoc analysis. DC, dendritic cell; ILC, innate lymphoid cell; BC, B cell; Treg, regulatory T cell; Th17, T helper 17 cell; TNFβ, tumor necrosis factor β; TLR4, toll-like receptor 4; NFκB, nuclear factor kappa light chain enhancer of B cells; Casp1, caspase1; IL-1β, interleukin 1 beta; CCL2(MCP1), monocyte chemoattractant protein 1; CCL5(RANTES), C-C motif 5; ICAM, intercellular adhesion molecule; VCAM, vascular cell adhesion molecule. In all cases, *p < 0.05, **p < 0.01, ***p < 0.001.

Mice were randomly subdivided into 8 treatment groups including untreated control (Control), BDPP-only (BDPP), probiotic or synbiotic and each treatment group was treated with one condition, either stressed or non-stressed. During the stress protocol, mice were submitted to 28 days of random mild stressors (CUS), allowed to rest for 28 days (CUS+Rest) followed by re-stimulation to a subthreshold mild unpredictable stress (US) for 7 days (CUS+US) to model recurrent stress. The CUS+US timepoint was controlled to a 7 day US without prior stress exposure as demonstrated in previous studied (**Figure 1A**) (15). For each treatment, condition (stressed or non-stressed) and timepoint, the group size included *n*=16 animals, combined over three independent experiments where tissues were allocated towards the biochemical and imaging studies equally. This group size was determined based on power analyses from previous data conducted by us (28) and others (15). During the stress periods, animals in all study groups, including the mice for the immunophenotyping, were subjected twice daily to random mild stressors (**Table S2**). Stressors included 45° cage tilt for 12 h, wet bedding for 10-12 h, no bedding for 10-12 h, food and/or water deprivation for 12 h, 4°C cold exposure for 1 h, cold water swim for 5 min, cage shaking for 20 min, reversed light schedule, restraint stress for 1 h, predator scent exposure for 8 h, or crowding with 12 animals/cage for 1 h. Consistent with our ongoing and published (28) experiments, no significant changes between groups were observed for weight, water or food consumption throughout the testing period. Behavioral assessment on all animals (*n* =16 per group) was conducted following each timepoint. From the 16 animals per group, 6 mice were perfused and used for immunofluorescent analysis, 6 mice were used for tissue RNA, protein (brain and peripheral tissues), blood and feces collection and the remaining 4 for other purposes not relevant to this manuscript. Animals were sacrificed immediately following behavior, blood was collected by cardiac puncture into heparinized tubes while tissues were frozen on dry ice and stored at -80°C until analysis.

### Behavioral Experiments

Behavioral experiments were performed with a NIR camera and measured with ANY-maze™ tracking software (Stoelting Co., IL, USA. Version 5.1 Beta). All animals were handled for 5 min per day for 3 days prior to behavioral testing and were habituated to the testing room for 1 h at the beginning of the test day. Details for anxiety-like behavior with the open field test and depressive-like behavior with the forced swim test can be found as previously described (28).

### 16S DNA Extraction and Gut Microbiome Profiling

Fecal pellets from six mice were isolated from the distal colon from 6 mice per group. The Qiagen PowerFecal Pro DNA kit (Cat No. 12830-50) was used for DNA extraction with the Omni International BeadRupter 24 bead mill homogenizer. DNA from extracted samples was amplified using Invitrogen’s AccuPrime High Fidelity kit using primers containing adapters for MiSeq sequencing and single-end barcodes allowing pooling and direct sequencing of PCR products (515F/806R) (33). The 16S rRNA gene sequencing was conducted by Diversigen (Houston, TX) (34). The 16S rRNA V4 gene region was amplified from the extracted community DNA by PCR and sequenced with the MiSeq platform (Illumina) using the 2x250 bp paired-end protocol. This generated paired-end reads that overlapped almost completely. Sequences that passed chimera slaying post sequencing were merged and blasted against the 16S specific curated Silva database (v132).

### RNA Extraction and Real Time PCR

Previous studies showed promising changes in immunological gene regulation in the ileum, spleen and multiple brain regions (28). To confirm these changes in the current study for multiple time points, RNA from the spleen and ileal tissues were extracted using the Trizol reagent (ThermoFisher) while RNA from brain regions was extracted using the RNeasy Mini kit (Qiagen), with the same *n* = 6 animals per group as used for the 16S sequencing. cDNA synthesis was conducted with the High-Capacity cDNA Reverse Transcription Kit with RNase Inhibitor (ThermoFisher). qPCR was conducted in collaboration with the Quantitative PCR CoRE at the Icahn School of Medicine at Mount Sinai using an ABI 7900HT Real-Time instrument and SDS software. Relative gene expression was assessed using the delta-delta CT method (35) and all primer pairs and annealing temperatures are provided in **Table S3**. All data was expressed in relative terms to the housekeeping gene *gapdh* following its demonstration of stability across tissue and treatment (36).

### Protein Extraction and ELISA Assays

Protein from brain regions, spleen and ileum from the same mice used for RNA and fecal collection (*n* = 6) were homogenized in RIPA buffer (Sigma) with an added protease inhibitor cocktail (Sigma) and phenylmethylsulfonyl fluoride (PMSF) phosphatase inhibitor (ThermoFisher). ELISA assays for the cytokines IL-1β (limit of detection, LOD 15.6 pg/ml, R&D Biosystems), IL-10 (31.2 LOD, R&D Biosystems), IL-6 (LOD 4.0 pg/ml ThermoFisher) and IL-17A (ThermoFisher, LOD 4 pg/ml) were conducted as per company instructions.

### Immunohistochemistry

At the time of sacrifice, mice (*n* = 6 per group, separate from the RNA, protein and fecal analysis) were cardiac perfused first with saline and then with 4% paraformaldehyde (PFA) and the brain including the brain stem was removed. Brain tissue was preserved in 4% PFA overnight and subsequently transferred to a saline solution containing sodium azide (0.02%). Sagittal sections were cut to 50 µm thickness using a vibratome (Leica VT1000S) and stored in saline with sodium azide. For immunostaining, slices were washed with PBST (PBS + 0.1% Triton X-100) and blocked in 5% normal goal serum. Slices were incubated with primary antibody (rabbit anti-mouse Iba1, clone EPR16588, abcam and rat anti-mouse CD68, clone FA-11, BioRad) and then secondary antibody (goat anti-rabbit AF568 and goat anti-rat AF488, ThermoFisher) before mounting with ProLong Diamond Antifade mounting media (ThermoFisher). Images were acquired on a Zeiss LSM880 Airyscan confocal microscope under an X20/0.8 NA air immersion 20 X objective. Image analysis was conducted with Image J. Microscopy image analysis was performed at the Microscopy CoRE at the Icahn School of Medicine at Mount Sinai.

### Single Cell Suspensions

Samples processed for CyTOF were derived from a different cohort of mice than the reported behavioral and biochemical analyses with *n* = 3 animals per group. Blood was drawn by cardiac puncture and placed in EDTA blood collection tubes on ice. PBMCs were extracted by serially lysing red blood cells (RBC) in RBC lysis buffer. The spleen and ileum (distal 5 cm of the small intestine) were removed and placed in ice-cold RPMI supplemented with FBS (2%) and HEPES (15 mM). The spleen was washed, macerated and placed in a digestion buffer containing RPMI supplemented with FBS (5%), DNase I (0.5 mg/mL) and collagenase IV (0.4 mg/mL) at 37°C for 30 min with agitation. After 30 min, the spleen was pulverized with a 18G needle and filtered through 100 µM mesh on ice. Ileum samples, with Peyer’s Patches removed, were washed in HBSS to remove fecal matter and cut longitudinally so mucus and remaining fecal matter could be removed. The whole ileum was placed in a dissociation buffer of RPMI supplemented with FBS (5%), EDTA (5 mM) and HEPES (15mM) for 20 min at 37°C with agitation. Following the 20 min, the dissociation buffer and ileum was filtered through a 100 µM filter the lamina propria was transferred to a digestion buffer of RPMI supplemented with FBS (5%), DNase I (0.5 mg/mL) and collagenase VIII (0.4 mg/mL) and incubated 25 min at 37°C with agitation. Like the spleen, the ileum was pulverized with a 18G needle and filtered through a 100 µM mesh on ice.

### GIPA02 CyTOF Sample Processing and Data Acquisition

Samples were processed by the Mount Sinai Human Immune Monitoring Center. Cell counts were performed on the Nexcelom Cellaca Automated Cell Counter (Nexcelom Biosciences, Lawrence, MA, USA) and cell viability was measured utilizing Acridine Orange/Propidium Iodide viability staining reagent (Nexcelom). Cell counts were normalized such that four million cells were taken for downstream processing. After washing cells, live-cell barcoding was performed. Live-cell barcoding allows for the pooling of samples prior to CyTOF antibody labeling, thus eliminating batch staining variability between replicates (37). In this study, cadmium conjugated CD45 and MHCI (H-2) antibodies were utilized for live-cell barcoding, and replicates of each condition were live-cell barcoded together. Fc receptor blocking (Biolegend Inc., San Diego, CA, USA) and Rhodium-103 viability staining (Fluidigm) were performed simultaneously with live-cell barcoding. Next, surface staining (antibody list **Table S4**) was performed by resuspending each pooled sample in a scaled amount of CyTOF antibody cocktail. Next, the eBioscience Foxp3/Transcription Factor Staining Buffer Set (Thermo Fisher Scientific, Waltham, MA, USA) was used to perform intranuclear staining. Samples were then washed and palladium barcoding of each condition was performed utilizing the Fluidigm Cell-ID 20-Plex Pd Barcoding Kit (Fluidigm) following manufacturer’s instructions. Conditions with unique combinatorial palladium labels were pooled such that only three samples remained (PBMC pool, spleen pool, ileum pool). This second tier of barcoding allowed intracellular staining to be performed on all of the conditions/replicates per tissue type in bulk. Heparin blocking (100 units/mL) was utilized to prevent non-specific binding of intracellular antibodies to eosinophils (38). Samples were fixed in 2.4% PFA. 125nM Iridium-193 (Fluidigm) and 2nM Osmium tetroxide (EMS) cell labeling was performed simultaneously with sample fixation (39). Samples were washed twice with CSB and stored in FBS + 10% DMSO at -80°C until acquisition (40).

Prior to data acquisition, samples were washed and resuspended at a concentration of 1 million cells per ml in Cell Acquisition Solution containing a 1:20 dilution of EQ Normalization beads (Fluidigm). The samples were acquired on a Helios Mass Cytometer equipped with a wide bore sample injector at an event rate of <400 events per second (41). After acquisition, repeat acquisitions of the same sample were concatenated and normalized using the Fluidigm software, and palladium-based debarcoding was performed utilizing the CyTOF debarcoding software made available from the Eli Zunder lab (42). These debarcoded files were uploaded to Cytobank for manual data clean-up/live-cell debarcoding.

For manual cleaning, immune cells were first identified based on Ir-193 DNA intensity and CD45 expression; Ce140+ normalization beads, CD45-low/Ir-193-low debris and cross-sample and Gaussian ion-cloud multiplets were excluded from downstream analysis. After this data cleanup, manual gating was utilized to debarcode the live-cell cadmium barcoded replicates. After this final debarcoding, the files were split by population such that each FCS file contained fully cleaned and debarcoded data from its corresponding sample. Lastly, for unbiased analysis of cell-subsets within each sample, the debarcoded files were run through the Astrolabe Diagnostics Data Processing pipeline (43). viSNE plots and cell type gating were preformed using Cytobank (44). viSNE clustering was performed on 22 parameters (**Table S5**) where equal event sampling was selected using 6666 events in the spleen, ileum and PBMCs, the lowest common denominator in all samples.

### MARS Algorithm and Correlation Analysis

All linear regression and Multivariate Adaptive Regression Splines (MARS) analyses were performed in R (version 4.0.2). The samples included were all derived from biological material from the same mice used for the 16S sequencing, RNA, protein and behavioral tests with *n* = 6 per group. Basic linear regression was performed in R using the lm function (“stats” R package, version 4.0.2). The association of individual bacterial species, gene expression and cytokines with subject behavior was examined with the following formula: log10(response) ~ Behavior + Day + Condition, where Day and Condition served as control variables. MARS analysis was performed in R using the “earth” package (version 5.1.2). The MARS approach allowed for the systematic identification of pertinent primary, secondary or higher levels of interactions among predictor variables. Briefly, MARS constructs a linear model with all possible basis terms (defined as hinge functions of predictors and the products of them), then by performing a stepwise term deletion in the full model, identifies the set of model terms that yield the best Generalized Coefficient of Variation (GCV). To assess the influence of microbiome on behavior, the MARS algorithm was run with the behavior as the response variable, while all available microbiome genera and conditions (i.e. time, stress or treatment) as predictors. To assess the influence of measured genes and cytokines on behavior, the MARS algorithm was run with the behavior as the response variable, while all available genes, cytokines, and conditions (i.e. time, stress or treatment) were predictors. The optimal parameters for MARS analysis, including the maximum degree of interactions, was determined using the generalized R-squared metric, which is the estimated model performance on unseen datasets. To systematically dissect which minor cell population was varying with respect to treatment and/or time, the MARS algorithm was repeated with the cell frequencies jointly as the predictor variable, and treating the timepoint, treatment and stress conditions as the response variable.

### Statistics

All statistical analyses were completed in Graphpad Prism version 8.0. All within group comparisons for the behavior, gene and cytokine data were conducted with 2-way ANOVAs with Tukey’s posthoc test while one-way ANOVAs with Tukey’s post-hoc analysis for the microglia activation marks and immune cell frequency data. For the gut microbiome analysis, between group statistics of the phyla and genera were conducted with the Mann-Whitney U test (due to the non-parametric nature of gut microbiome data) with false discovery rate (FDR) corrections using the Benjamini-Hochberg method. Alpha diversity, representing microbiome variance within a sample, was calculated with the Fisher diversity index and Observed OTU abundances. Beta diversity, between sample variance, was estimated quantitatively with weighted UniFrac analysis distance matrices. Variation in community structure was assessed with permutational multivariate analyses of variance (PERMANOVA) between groups with visualization of the data using Principal Coordinates Analysis (PCoA).

## Results

### A Synbiotic Attenuates Chronic and Recurrent Stress-Induced Behavioral Impairment

Chronic and recurrent stress were modelled using the CUS paradigm (**Figure 1A**). This paradigm was used to elucidate the gut microbiome-peripheral-brain-inflammation connection in driving chronic- and recurrent-stress induced behaviors. As previously shown, behavioral changes were not observed following US (15); however, significant depressive- (**Figure 1B**) and anxiety-like (**Figure 1C**) behaviors were observed in the stressed vehicle controls following CUS. Both behavioral impairments recovered following the rest period (CUS+Rest) and were re-observed with CUS+US re-stimulation. The probiotic and synbiotic attenuated depressive-like behavior compared to stressed vehicle controls following CUS, while the synbiotic rescued the phenotype following CUS and CUS+US (**Figure 1B**). All treatment groups elicited a beneficial effect on anxiety-like behavior following CUS and CUS+US compared to the stressed vehicle control (**Figure 1C**). Notably, only BDPP and the synbiotic, however, improved anxiety-like behavior compared to their respective unstressed controls at the CUS timepoint. Taken together, the synbiotic promoted more consistent and robust resilience to stress-induced behavioral impairments compared to its components.

### Neuroinflammation in Limbic Brain Regions Do Not Associate With Synbiotic-Induced Behavioral Phenotypes in Chronic and Recurrent Stress

**Figure 2:**
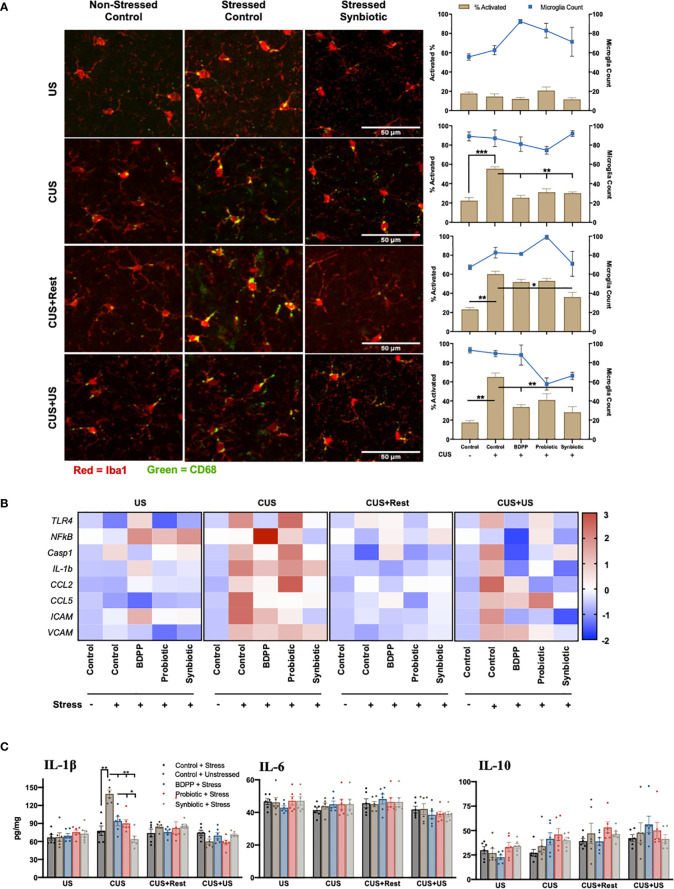
Dissimilarity between recurrent stress behavioral phenotype with neuroinflammatory correlates. The behavioral phenotype was aligned with microglia activation in the prefrontal cortex as determined by **(A)** immunohistochemical analysis of Iba1 (red) and CD68 (green) for area and activation, respectively (*n* = 6 +/- SEM). Quantification was determined as the percentage of surface area covered by CD68 *vs.* Iba1 (brown bars, left axis) and displayed with the microglia count per frame (blue line, right axis) with significance determined with one-way ANOVA and Tukey’s post-hoc analysis. **(B)** Immunological markers of microglia activation were validated with region-specific gene expression (*n* = 6, different mice than used for the IHC) and represented as the ratio of stressed *vs.* non-stressed for the respective group normalized with the z-score across all timepoints for a single gene. A positive z-score is represented with red and a negative z-score with blue. Absolute quantification of key cytokine expression **(C)** of IL-1β (left), IL-6 (middle) and IL-10 (right). Statistical markers are delineated by *p < 0.05, **p < 0.01 and ***p < 0.001.

Studies have shown that microglia activation in limbic brain regions mirror behavioral responses of chronic and recurrent stress (15). In this study, microglia activation was determined by the activated CD68 positive surface area. There were no variations in microglia activation or number in the prefrontal cortex (PFC) with US, but following CUS, microglial activation was elevated in the stressed vehicles and attenuated by all treatments (**Figure 2A**, **Figure S2**, **Table S7**). Notably, microglia activation remained elevated at the CUS+Rest and CUS+US timepoints in discordance with behavioral observations while the synbiotic ubiquitously attenuated activated microglia at all time points. A similar analysis was done in the amygdala, hippocampus and nucleus of the solitary tract (NTS) of the cerebellum. In the amygdala, a similar trend was observed with respect to timepoint and treatment, while there were few inconsistent changes in both the hippocampus and the NTS (**Figures S1**, **S2**, **Tables S8**–**13**). To test if microglial activation could be reflected by transcriptional control of immune genes, quantification of key sterile immune factors, canonical activators and immune cell recruitment genes in the respective brain regions was conducted. Following CUS and CUS+US, there was an upregulation of almost all immune-associated genes including toll-like receptor (*Tlr*)4, caspase 1 (*Casp1*), *Il1β*, *Ccl2*, *Ccl5*, intercellular adhesion molecular (*Icam*) and vascular adhesion molecule (*Vcam*) in the PFC (**Figure 2B**, **Table S4**). At the gene level, the synbiotic, but not the probiotic or BDPP, robustly attenuated inflammatory gene expression profiles. Importantly, the most consistent and drastically impacted factors by both stress and the synbiotic, paralleling the behavioral response, were the immune cell recruitment genes, *Ccl2*, *Ccl5* and *Icam.* In the PFC, their expression in response to stress and treatment mirrored the behavioral response with the synbiotic attenuating their stress-induced elevation. This was similarly true for the gene and cytokine expression in the PFC, hippocampus, cortex and cerebellum, however less defined compared to the PFC (**Figure 2C**). This suggests that the recruitment of peripheral immune cells into the brain may be more important than microglia activation for driving the gut-microbiota stress-induced behavioral responses.

### Gut Microbiota Variations Under Chronic and Recurrent Stress Normalized by Synbiotic Treatment

**Figure 3:**
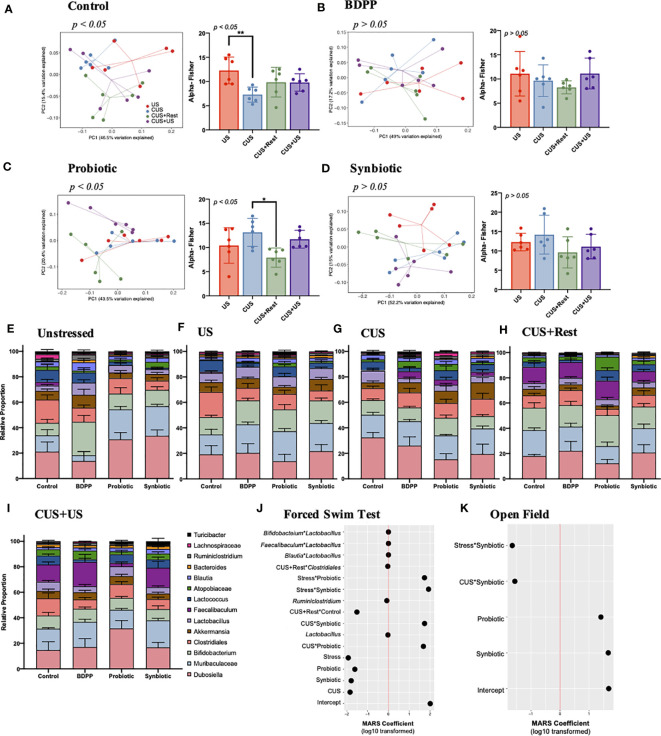
Chronic and recurrent stress induced variations in the gut microbiota in response to synbiotic treatment. 16S V4 metagenomic sequencing of the gut microbiota following chronic and recurrent stress and treatment with variations in genera across all timepoints in the CUS protocol: US, CUS, CUS+Rest and CUS+US. Variation in community structure was assessed with permutational multivariate analyses of variance (PERMANOVA) between groups (beta diversity) with visualization of the data using Principal Coordinates Analysis (PCoA) with the lines representing the distance of each individual to the centroid. The alpha diversity calculated as the Fisher’s Alpha test following **(A)** stressed vehicle control, and stress with **(B)** BDPP, **(C)** Probiotic and **(D)** Synbiotic. Statistical significance is marked by *p < 0.05 and **p < 0.01. Variations in the gut microbiota over time, within treatment group can be found in **Figure S3**. Variations in the 14 most abundant genera with respect to treatment is shown in **(E)** unstressed vehicle controls, **(F)** US, **(G)** CUS, **(H)** CUS+Rest and **(I)** CUS+US with statistical differences outlined in **Figure S4**. Results from the MARS algorithm show the interactions of gut microbiome and study covariates predicting **(J)** forced swim test and **(K)** open field test. The x-axis depicts the signed log10 transformed absolute value of the term coefficient in the MARS model. Note that the hinge function was removed for simplicity of visualization. Each group contains *n* = 6 mice (the same mice used for RNA/protein studies) +/- SEM individuals while significance determined with one-way ANOVA with Tukey’s posthoc analysis. Statistics for the β-diversity is as outlined in material and methods section.

The gut microbiome is sensitive to both stress and treatment and plays a critical role in directing the immune response along the gut-brain-axis (2). To determine how the synbiotic modulates the gut microbiome’s response to the stress protocol, 16S metagenomic sequencing was conducted. The stress protocol caused a significant shift in both the Fisher alpha (within sample variance) and beta diversities (between sample variance) in the gut microbiome of stressed vehicle controls (**Figure 3A**). The stress-induced variations in alpha and beta diversities were normalized by BDPP (**Figure 3B**) and the synbiotic (**Figure 3D**), but not probiotic treatment (**Figure 3C**). Based on these results, the chronic stress-induced variation following CUS can be interpreted as a loss of microbial diversity, which partially recovered after a period of rest. Note, that the alpha diversity was unchanged in unstressed treated controls; however all treatments elicited a similar shift in the beta diversity (**Figure S3A**). Likewise, treatment affected beta diversity following US and CUS (**Figures S3B, C**), while alpha diversity varied only following CUS due to an increase in diversity observed with probiotic and synbiotic treatment (**Figure S3C**). Interestingly, treatment had no effect on the alpha or beta diversities following CUS+Rest or CUS+US (**Figures S3D, E**). Taken together, the synbiotic was the most effective at normalizing the changes in gut microbiota diversity due to chronic and recurrent stress.

Quantification of the individual gut microbiome populations confirmed the consistent beneficial role of the synbiotic. There were no variations in either the phyla (**Figure S3F**) or genera (**Figure 3E**) of non-stressed treated controls due to either stress or treatment; however, both treatment and time elicited variations in the genera of stressed mice (**Figures 3F–I**, **S5**). The most drastic variation following CUS in the stressed vehicles was an increase in *Dubosiella* spp., which recovered after the rest period but failed to increase upon CUS+US (**Figure S4**). Following CUS, both probiotic and synbiotic treatments effectively reduced *Dubosiella* spp. levels (**Figure S5**), while the synbiotic attenuated *Dubosiella* levels at all time points (**Figure S4**). *Faecalibacterium* spp. is related to *Dubosiella* spp. yet there were no variations in *Faecalibacterium* spp. in stressed vehicle controls at US or CUS in contrast to the increase observed following CUS+Rest and CUS+US (**Figure S4**). This trend was paralleled by all treatment groups (**Figure S4**); however, the synbiotic elicited a proportionally larger increase in *Faecalibacterium* spp. than the other treatments (**Figures 3F**, **S5**). *Akkermansia* spp. is another important genera in terms of metabolite production, but there were no variations due to stress in vehicle controls at any timepoint (**Figure S4**). Importantly, the synbiotic alone elicited a positive increase in *Akkermansia* spp. following CUS compared to stressed vehicle controls (**Figures 3F**, **S5**). Finally, the probiotic and synbiotic elicited a stark increase (and BDPP a decrease) in *Lactococcus* spp. at all time points, compared to the non-stressed vehicle controls (**Figure S4**), yet there were no variations in *Lactococcus* spp. due to stress or timepoint in vehicle controls. Overall, the probiotic and synbiotic treatments normalized the major genera variations induced by stress in vehicle controls while the synbiotic elicited some unique changes in genera populations that play important roles in managing barrier integrity.

To understand how variations in the gut microbiota, and their interactions, may relate to the behavioral response in the context of chronic and recurrent stress, a MARS model was performed with behavior as the response and all available microbiome genera and conditions (i.e. time, stress or treatment) as predictors. In this context, the MARS algorithm takes into account the variations of all the gut microbiota populations, group and timepoint effect and the combined behavioral data to unbiasedly predict which groups of bacteria influencing the overall phenotype of chronic-stress induced behavioral impairment. The use of the MARS approach allows for the systematic identification of primary and secondary levels of interactions between gut microbiome populations, which optimize the prediction of stress-induced behavioral responses. It also controls the individual covariates’ influence (i.e. time, stress or treatment) on the behavioral response based on the microbiome data. Depressive-like behavior associated with variations in *Lactobacillus* spp., *Ruminoclostridium* spp., and secondary interactions of *Lactobacillus* spp. with *Faecalibaculum, Blautia*, or *Bifidobacterium* spp. (**Figure 3J**). While the log10 transformed MARS coefficients appear very small, these interactions remain significant considering the extensive size of the model with multiple covariate influences. With a larger coefficient, the interaction of stress with either probiotic or synbiotic imposed a positive effect on the depressive-like behavior (**Figure 3J**). In contrast, anxiety-like behavior did not associate to any specific genera (**Figure 3K**), but synbiotic and probiotic treatment did associate to anxiety-like behavior. Additionally, the interaction of the synbiotic with stress or the CUS timepoint both associated to lower anxiety demonstrating the beneficial effect of the synbiotic. These results indicate that probiotics and synbiotics, but not BDPP, alter the gut microbiota in a manner correlating to attenuated stress-induced anxiety- and depressive-like behavior, while depressive-like behavior is more sensitive to specific gut microbiota genera including *Lactobacillus* spp. Further studies will be required to understand the direct relationship between genera specially altered by the synbiotic and the direct psychological consequences.

### A Synbiotic Protects Against Intestinal Inflammation

**Figure 4:**
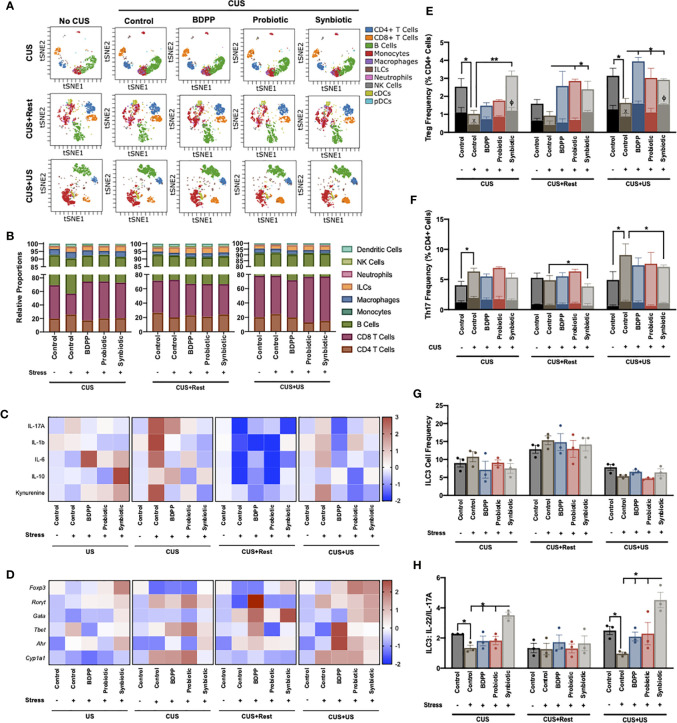
Synbiotic alleviates ileal immune variations in response to chronic and recurrent stress. Immunophenotyping of the ileum was determined with a CyTOF panel and **(A)** overall visualization of the similarity of major cell populations in a single representative sample is depicted as a viSNE plot. The viSNE plot is two-dimensional figure with the axes tSNE1 and tSNE2, with cells plotted on a continuum of expression with phenotypically related cells clustered together based on gating of major cell populations and colored based on cell type called a “phenotypic island”. Quantification of each of these cell populations (*n* =3) is shown in **(B)**. Overall immunological response is depicted as a heatmap of the **(C)** cytokine and **(D)** transcriptional profiles (*n* = 6) and represented as the ratio of relative values of stressed *vs.* non-stressed for the respective group normalized with the z-score across all timepoints for a single gene or cytokine. A positive z-score is represented with red and a negative z-score with blue. Individual cell frequencies of **(E)** regulatory T cells (Tregs) and **(F)** T helper (Th) 17 cells relative to total CD4^+^ cell populations are shown with a solid color inset of activated Treg (CTLA^+^) and Th17 (IL17A^+^ICOS^+^) cells, respectively. Frequency of **(G)** innate lymphoid cell (ILC)3 relative to total cell populations and **(H)** the ratio of IL-22 to IL-17A producing ILC3 cells are shown. All cell frequencies represent *n* = 3 mice +/- SEM with significance determined with a one-way ANOVA and Tukey’s post-hoc analysis where *p < 0.05, **p < 0.01.

Chronic stress and variations in the gut microbiota are intimately related to barrier immunity and programming of peripheral cellular immune responses. Further, the behavioral responses to stress and synbiotic treatment were more sensitive to the recruitment of peripheral immune cells to the brain than neuroinflammation. To characterize the peripheral innate and adaptive immune cell variations in response to stress and treatment, immunophenotyping was conducted with CyTOF. In the ileum, viSNE analysis gated by the major cell populations (**Figure 4A**) and the corresponding quantification of the major cell types (**Figure 4B**) revealed that neither treatment nor time elicited significant variations in the major ileal immune cell populations. Note that the viSNE plots were calculated for each timepoint so direct comparisons of the phenotypic islands cannot be compared between timepoints. Continuing broad characterization of the ileal immunological response, the gene and cytokine expression of key inflammatory components were determined. Following CUS and CUS+US, but not US or CUS+Rest, there was a strong upregulation of kynurenine and the proinflammatory cytokines in the ileum including IL-17A, IL-1β and IL-6, an effect rescued most strongly by the synbiotic (**Figure 4C**, **Table S5**). There was a correlating downregulation of the anti-inflammatory cytokine IL-10 in control mice in response to stress, while the synbiotic ubiquitously up-regulated IL-10 release. Notably, unlike the major cell populations, cytokine expression mirrored the stress-induced behavioral responses. A similar trend was observed for gene expression. US invoked few variations in immunological gene expression, but CUS and CUS+US strongly upregulated *Ror*γ*t* and *Cyp1a1* in stressed vehicle controls, which were both downregulated exclusively by the synbiotic following CUS, but not CUS+US (**Figure 4D**, **Table S6**). The gene expression ratio of *Ror*γ*t* to *Foxp3*, reflecting broadly the Th17 to Treg ratio, was upregulated by CUS and CUS+US in the stressed vehicle controls, and rescued by the synbiotic following CUS and all treatments following CUS+US (**Figure S6A**). This trend was similar to the ratio of IL-17A to IL-10 cytokine release (**Figure S6B**) indicating that the variations in proinflammatory gene expression elicited by stress and beneficially affected by the synbiotic may be due to the regulation of Th17 and Treg cell differentiation.

Enumeration of Tregs and Th17 cells and their activated states was determined using the CyTOF data. In stressed vehicle controls, ileal Tregs were reduced following CUS and CUS+US, an effect ubiquitously upregulated by the synbiotic, CUS+US by BDPP and CUS+Rest and CUS+US by probiotics (**Figure 4E**). Interestingly, CTLA4^+^ Tregs (i.e. activated Tregs) were correspondingly decreased following CUS and CUS+US in stressed vehicle controls and elevated at all timepoints exclusively by the synbiotic (**Figure 4E**). A different trend was observed for the Th17 cells. Th17 cell numbers were elevated following CUS and CUS+US in the stressed vehicle controls, but only downregulated by the synbiotic in the CUS+US group (**Figure 4F**). Likewise, there were no significant changes in the IL-17A^+^ICOS^+^ Th17 cells (i.e. activated Th17) with respect to time or treatment. Concatenating this, the ratio of inactivated Th17/Treg cells in the ileum was significantly elevated in stressed vehicle controls following CUS and CUS+US, compared to unstressed vehicle controls. This stress-induced elevation was ubiquitously rescued only by the synbiotic at all time points (Fig S6c). A similar trend was observed for the ratio of “activated” Th17/Treg, except the synbiotic exclusively reduced the activated Th17/Treg ratio except at the CUS+US timepoint where probiotics also elicited a beneficial effect (**Figure S6C**). These trends were unique to the Treg and Th17 cells, as the other effector lymphocytes in the ileum including CD8 T cells (IFN*γ*+ and TNFα+), Th1 and Th2 cells showed inconsistent variations with respect to time and treatment (**Figure S7**, panels within).

To understand which innate immune cells may be activating the lymphocytes in the context of gut microbiota-inflammatory-stress interactions, several APCs were also assessed in the ileum. The quantities of monocytes, macrophages, dendritic cells (DC1, DC2, pDC), natural killer (NK) cells and neutrophils showed little variation with respect to either time or treatment (**Figure S7**, panels within). As reported in other studies, there was an increase in MHCII^+^ monocytes in the ileum of stressed vehicle controls following CUS and CUS+Rest, which was reduced by all treatments (**Figure S7C**); however, there was no effect following CUS+US. Interestingly, ILC, ILC1, ILC2 (**Figure S7**) and ILC3 (**Figure 4G**) populations elicited few variations over time and treatment; however, the IL-22^+^ (NCR^+^) and IL-17A^+^ (NCR^-^) producing ILC3 cells showed trends paralleling the behavioral responses. IL-22 producing ILC3s were significantly downregulated in response to stress in vehicle controls, while synbiotic treatment exclusively upregulated their expression (**Figure S7J**). Likewise, synbiotic treatment downregulated the stress-induced increase in IL-17A producing ILC3 cells (**Figure S7K**). Overall, the ratio of IL-22/IL-17A producing ILC3s was reduced in response to stress and rescued exclusively by the synbiotic at both the CUS and CUS+US timepoints (**Figure 4H**). This is a significant result as IL-22 and IL-17A producing ILC3s are, in part, responsible for the differentiation of either Treg or Th17, respectively (45). It also suggests that manipulation of the gut microbiota with a synbiotic may reprogram the barrier immune architecture towards an NCR+ ILC3-Treg dominating phenotype that consequently prevents the stress-induced inflammatory milieu associated with chronic and recurrent stress.

### Crosstalk Between Peripheral Tissues Predict Stress-Induced Behavioral Responses

**Figure 5:**
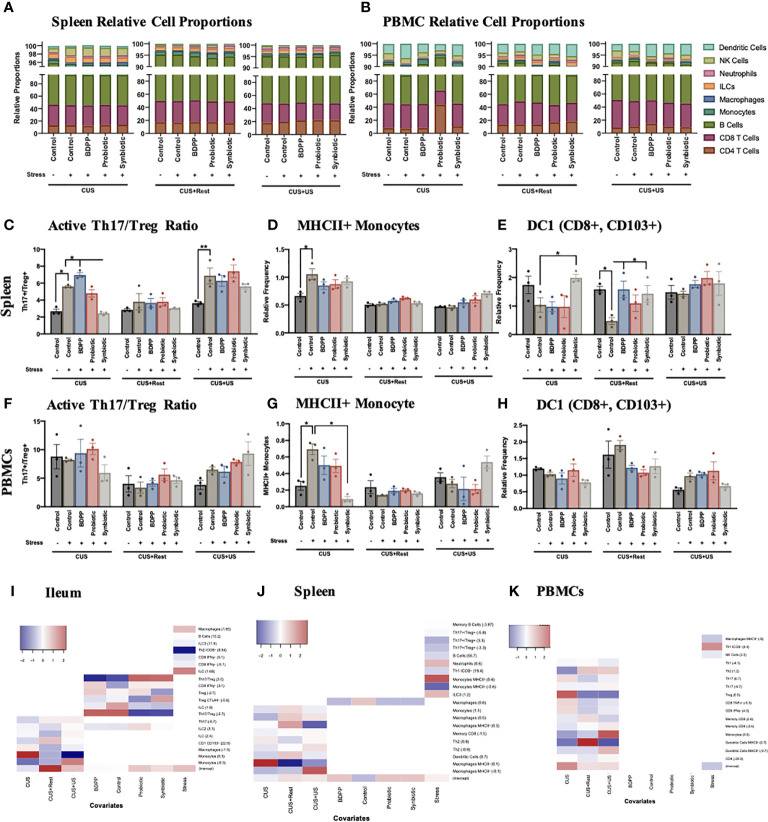
Interaction between peripheral and central immune cell profiles determine stress-induced behaviors. Immunophenotyping of the major cell types in the **(A)** spleen and **(B)** PBMCs show little variation across time during the CUS protocol, which is also reflected in the viSNE plots (**Figure S9**). Splenic cell frequencies reflect the activated **(C)** Th17/Treg ratio, **(D)** MHCII+ monocytes and **(E)** migratory CD103+ dendritic cells (DC)1 relative to total cell populations. PBMC cell frequencies of **(F)** Th17/Treg ratio, **(G)** MHCII+ monocytes and **(H)** migratory CD103+ dendritic cells (DC)1 relative to total cell populations are also shown. All cell frequencies have *n* = 3 mice +/- SEM with significance calculated with a one-way ANOVA and Tukey’s post-hoc analysis where *p < 0.05 and **p < 0.01. The cellular frequencies were associated to the individual study predictors (stress, timepoint and treatment) using a MARS additive model in the **(I)** ileum, **(J)** spleen and **(K)** PBMCs with the model hinge function in parentheses. The MARS term coefficient is represented as a heatmap with the higher coefficient represented with red color and the lower coefficient with blue.

To expand on the immunophenotyping of the ileum and gather a full understanding of the microbiota-peripheral-brain immune crosstalk, innate and adaptive immune cells in both the spleen (**Figure 5A**, **Figure S8A**, **Tables S14**, **15**) and PBMCs (**Figure 5B**, **Figure S8D**, **Tables S16**, **17**) were also immunophenotyped. In the spleen, stress-induced alterations in the Th17/Treg activated cell ratio was similar to the ileum, except the synbiotic elicited a less drastic impact (**Figure 5C**), which can be attributed to variations in the Treg population (**Figure S9G**). Variations in the Th17/Treg ratio were not observed among the PBMCs (**Figure 5F**). There was also an increase in MHCII^+^ monocytes in the spleen following CUS in the stressed vehicle controls (**Figure 5D**) and a trending decrease of migratory CD103^+^ DC1 cells, where the latter was rescued by the synbiotic following CUS and CUS+Rest (**Figure 5E**). There was a similar increase in activated monocytes among PBMCs following CUS in the stressed vehicle control, an effect rescued by synbiotics alone (**Figure 5G**); but no alterations due to stress or treatment were observed in the migratory DC1 population (**Figure 5H**).

Gene and cytokine expression previously shown to be altered by stress and synbiotic treatment in the periphery (28), showed less consistent variations in the context of chronic and recurrent stress. There was a stark increase in *Ror*γ*t, Cyp1a1* and *Tlr4* in the splenic stressed vehicle controls following CUS, all rescued by synbiotic treatment (**Figure S8B**). Importantly, these variations were not observed in the CUS+US group uncoupling the inflammatory response in the spleen from the behavioral response. A similar trend was observed for splenic cytokine expression (**Figure S8C**). In the serum, the proinflammatory cytokines IL-1β and IL-17A were upregulated following both CUS and CUS+US in the stressed vehicle controls, and downregulated by the synbiotic (**Figure S8E**). Notably, IL-17A expression remained elevated following rest (CUS+Rest) in the stressed vehicle controls, also uncoupling it from the behavioral phenotype. Importantly, unlike in the ileum, the variations in immune cells observed in the PBMCs and spleen generally did not associate with the chronic and recurrent stress-induced behavioral impairment, yet a trend towards a chronic-stress induced peripheral immune cell phenotype may be apparent. As such, in both the spleen (**Figure S9**) and PBMCs (**Figure S10**), variations in minor cell populations were observed, yet not obviously correlated to the behavioral phenotypes.

To systematically dissect which minor cell populations were varying with respect to treatment and/or time, the MARS model was repeated, using an additive model, with the cell frequencies as the predictors to the timepoint, treatment and stress as responses. As these data were collected from a separate cohort of animals not used for the above behavioral analyses, exploration of a link between cell frequencies and behavior was not attempted. This analysis will determine which of the cell frequencies are associated to the study conditions, i.e. day, stress or treatment. In the ileum, the Th17/Treg ratio was positively associated to the control and BDPP treatments, while negatively associated to probiotic and synbiotic, defining the beneficial effect specifically of the probiotic and synbiotic on this lymphocyte ratio within the context of chronic and recurrent stress (**Figure 5I**). A similar trend was observed for the activated CTLA4+ activated Tregs. Regarding the variation associated to timepoint (CUS, CUS+Rest or CUS+US), the monocytes, macrophages and dendritic cells all displayed significant associations. This apparent dichotomy between cell types responsive to timepoint (mostly APCs), cell types associated to treatment (lymphocytes and ILCs) and cell types varying with stress demonstrate a clear separation of regulatory mechanisms to be considered in the ileum of the current stress and treatment model.

A similar separation was observed in the spleen and PBMCS. In the spleen, there was a strong association of the immune cell subsets with the timepoint, while only macrophages weakly associated with treatment (**Figure 5J**). In addition, and in support of the cell frequency quantification, MHCII^+^ monocytes were highly associated with the overall stress condition, whereas MHCII^+^ macrophages more strongly associated to the individual timepoints. Finally, cell subsets in stressed animals significantly varied with respect to timepoint in the PBMCs, not treatment and only weakly with stress (**Figure 5K**). The strongest association in PBMCs was the activated MHCII^+^ dendritic cells and the Tregs, which were both responsive to timepoints associated with stress. This analysis revealed that there is an immune cell tissue-dependency in the context of chronic and recurrent stress, with the ileum having a considerable association to both time and treatment and in the spleen and PBMCs, the associations are primarily with the timepoint.

### A Peripheral-Central Immune Crosstalk Drives Chronic- and Recurrent-Stress Behavioral Deficits

**Figure 6:**
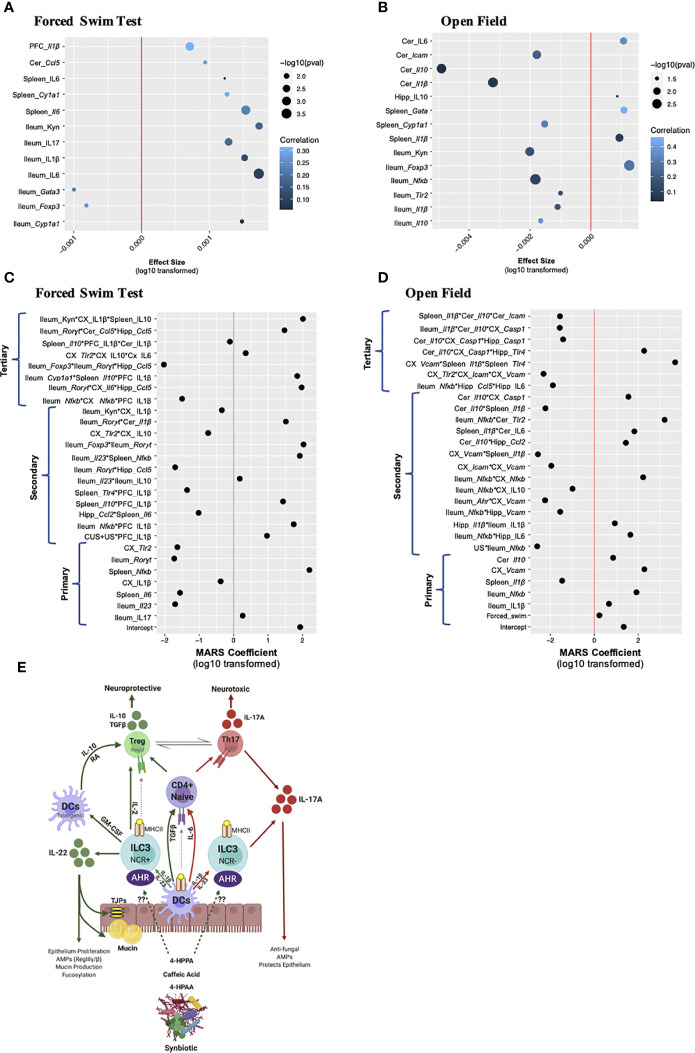
Multivariate analysis of gut-brain-axis coordination towards chronic- and recurrent-stress behavioral predictions. Linear regression modeling in which the individual gene, cytokine, and microbiota were used as response variables, and the behavior was used as the main predictor of interest, while condition, treatment and timepoint were considered covariates. Association to **(A)** depressive-like behavior and **(B)** anxiety-like behavior are shown with the x-axis representing the effect size, the y-axis the term and the significance shown as dot size and correlation (i.e. how well the model fits the response variable) as a heatmap. The x-axis represents the absolute value of the response variable log10 transformed, and multiplied by the sign, to establish a normal distribution: i.e. log10(effectsize+1) when the effect size was positive, and log10(abs(effectsize-1))*(-1) when the effect size was negative. A multivariate adaptive regression splines (MARS) model was designed to test the cross-tissue association of gene and cytokine factors (up to tertiary interactions) in the context of predicting **(C)** depressive-like behaviors and **(D)** anxiety-like behaviors. The x-axis shows the log10 of the absolute value of the coefficient, multiplied by the coefficient sign. For both the linear and MARS models, factor names in italics represent mRNA gene expression while normal text is cytokine expression. **(E)** A working scheme of barrier immunity affected by chronic and recurrent stress and the role of the gut microbiota and its associated metabolites. For all MARS associations, data from the same *n* = 6 mice were included from the 16S sequencing, RNA, protein and behavioral analyses. DC, dendritic cell; ILC, innate lymphoid cell; NCR, natural cytotoxicity receptor; AHR, aryl hydrocarbon receptor; RA, retinoic acid.

The peripheral, especially ileal, immune response is an important predictor of chronic and recurrent-stress induced behavioral impairment; however, neuroinflammatory markers in limbic brain regions are common signatures of stress-induced depressive- and anxiety-like behaviors. To dissect which peripheral and/or brain-derived immune factors are associated to the chronic and recurrent stress-induced behavioral responses, linear and multivariate interactions between immune factors and the behavioral response were made. Data was only included which was collected from the same animal, allowing only the 16S sequencing, behavioral, RNA and protein enumerations to be included. Linear associations between the gene and cytokine factors with the behavior response were conducted, with the gene and cytokines acting as the response variable and the behavior as the main predictor. In this model, the stress, timepoint and treatment were all treated as covariates giving an overall indication of the how the behavioral response associates with single tissue-specific immune genes and cytokines. Note, that the effect size represents the strength of the association while correlation represents the overall fit of the model in the context of all the covariates. In both cases where the forced swim test (**Figure 6A**) or the open field test (**Figure 6B**) were used as the predictor, one striking observation was that there were more peripheral immune associations compared to brain-derived ones. Predicting depressive-like behavior, only *Il1β* gene expression in the PFC and cerebellar *Ccl5* elicited a positive effect size, with the PFC response having a higher correlation than the cerebellum, indicating that the entire model fit well for the behavioral prediction. Splenic IL-6 (gene and cytokine) and *Cyp1a1* also displayed positive effect sizes with splenic *Cyp1a1* having a strong correlation demonstrating the importance of AHR pathway signaling. The remaining seven associations occurred in the ileum with the proinflammatory markers kynurenine, IL-17, IL-1β and IL-6 having positive and *Foxp3* and *Gata3* gene expression negative effect sizes (**Figure 6A**). Predicting anxiety-like behavior, which has an opposite direction of beneficial effect compared to the forced swim test (i.e. “lower” value in the open field test means less anxiety-like behavior), cerebellar IL-6 had a positive effect size and good correlation while *Icam, Il10* and *Il1β* all had negative effect sizes and weak correlations (**Figure 6B**). Similar to the forced swim test data, splenic *Cyp1a1* had a negative effect size and *Gata3* gene expression a positive effect size and strong correlation to the model. Also similar to the depressive-like behavior, was the dominant representation of ileal immune associations. Again, *Foxp3* had a strong response to anxiety-like behavior in the ileum with an opposing effect size compared to the other proinflammatory markers such as kynurenine, *Nfkb, Tlr2, Il1β* and *Il10* (**Figure 6B**). Overall, the linear model confirms that the peripheral, namely ileal, immune responses are better associated than the limbic brain-derived immune factors to the chronic and recurrent stress-induced behavioral response within the context of gut microbiota modifying prebiotics, probiotics and synbiotic suggesting that a direct relationship may exist.

Linear associations give valuable insights to the impact of the behavioral response on a single gene or cytokine predictor; however, this model is limited as the genes and cytokines are assumed to be independent, which could contribute to the positive association or explain the low associative coefficient. To detangle these covariates and predict which individual factors across tissue, treatment and time may be synergizing in response to the behavioral output, the MARS approach was implemented. Similar to the linear model, behavior [either depressive-like (**Figure 6C**) or anxiety-like (**Figure 6D**)] was used as a response variable, while all available genes, cytokines and sample characteristics (timepoint, stress, and treatment) were used as predictors. Using the generalized R-squared as criterion, allowing a maximum of three interactions in a single term provided a better model fit than when a maximum of two interactions were allowed (see details in materials and methods section). Using this analysis, primary, secondary and tertiary interactions can be identified. Similar to the linear associations, the ileum had the highest predictive power, with genes (*Ror*γ*t, IL-23R* and *Nfκb*) and cytokines (IL-17A and IL-1β) having significant primary associations in both behaviors tested. For the spleen, gene expression of *Il6, Il1β* and *Nfκb* likewise associated with behavior. In the brain, IL-1β in the PFC associated with depressive-like behavior whereas *Vcam, Tlr2* and IL-1β in the cortex and *Il-10* in the cerebellum also had significant primary effects.

The tertiary interactions bring a novel understanding to the capacity of the entire gut-brain-axis to drive chronic and recurrent stress-induced depressive-like behaviors synergizing across the tissues investigated. Interestingly, the triple association between ileal *Foxp3, Ror*γ*t* and hippocampal *Ccl5* strongly associated with depressive-like behavior (**Figure 6C**) linking the Th17/Treg ratio to the recruitment of peripheral immune cells to the hippocampus. Likewise, ileal *Ror*γ*t*, cerebellar *Ccl5* and hippocampal *Ccl5* were a strong predictor of depressive-like behavior as was ileal *Cyp1a1*, splenic *Il10* and PFC IL-1β. Ileal *Nfkb* expression also coordinated with hippocampal *Ccl5* and IL-6 production to predict anxiety-like behavior (**Figure 6D**). A splenic increase in *Il1β* and *Tlr4* with cortical *Vcam* expression strongly associated with the anxiety-like behavior while splenic IL-10 associated with IL-1β from both the PFC and cortex, predicted depressive-like behavior. These and the other associations illustrate how the gut-brain-axis may functionally coordinate with the limbic brain regions to predict stress-induced depressive- and anxiety-like behavior outlining the importance of the gut microbiota and barrier immunity in driving these responses. These correlations set the foundation for further studies to directly validate the impact of these singular and synergistic associations using transgenic mice and specifically immuno-compromised mice.

## Discussion

Chronic and recurrent psychological impairment are causally associated with inflammation (9, 46); however, the role of barrier immunity and the impact of gut microbiota on the peripheral and neuroinflammatory biological signatures of chronic and recurrent stress remain undefined. This is surprising as gut-brain associations were previously shown with studies outlining comorbidities between irritable bowel syndrome and psychiatric disorders including depression (47) and the benefit of probiotics for managing both the gastrointestinal and psychiatric symptoms (48). The gap in understanding for the role of barrier and peripheral immunity on the biological signature of chronic- and recurrent-stress induced psychiatric disorders formed the motivation of this study: to characterize the peripheral immunological changes elicited by a synbiotic that prophylactically prevents chronic and recurrent psychiatric symptoms associated with stress.

Chronic stress alters the commensal microbiota in mice (49) and humans (50). A dysbiotic microbiota has also been associated with MDD (51) and PTSD (52); however, neither functional nor causal links have been made between the gut microbiota and the symptoms associated with chronic or recurrent stress. We show that the diversity and complexity of the gut microbiota is reduced with chronic stress, which recovers following a period of rest yet does not recur following subthreshold recurrent stress. Despite this behavioral discordance, synbiotic treatment prevented the loss of diversity and complexity due to the stress protocol. Although there were no significant changes in phyla due to stress or treatment, *Dubosiella* spp. were highly upregulated following chronic, but not recurrent, stress, which was rescued by both probiotic and synbiotic treatment. *Dubosiella* spp. is composed largely of the species *Dubosiella newyorkensis*, which is closely related to *Faecalibaculum rodentium* (53). This novel genera was identified a part of the family *Erysipelotrichaceae* known to be important for host metabolism and inflammatory conditions associated with diet (54). *Erysipelotrichaceae* family members have been identified as “colitogenic strains”, which are heavily coated in IgA antibodies, can transfer colitis-like symptoms through cohousing and associate with inflammasome-mediated intestinal dysbiosis (55, 56). These are significant associations as they confirm how the *Dubosiella* spp. may influence the CUS-induced behavioral phenotypes by driving intestinal inflammation. Since the synbiotic at all timepoints reduced *Dubosiella* spp. populations, it demonstrates how a synbiotic proves superior to its probiotic or prebiotic constituents to regulate the commensal gut microbiome populations, protecting it against an inflammatory microbiome.

Chronic and recurrent stress invoked several minor gut microbiome alterations with respect to treatment and timepoint and a machine learning MARS algorithm was used to dissect which species were associated with chronic and recurrent stress-induced psychiatric impairment, in the context of time and treatment covariates. The MARS algorithm is a powerful approach as it considers all the variations occurring simultaneously between multiple data, groups, treatments and timepoints taking into the consideration the variations of each of the individual animals tested. The current study produces a large dataset across tissue, treatment, time and modality to gather enough information to make accurate predictions. This unbiased approach allows educated associations to be built facilitating mechanistic conclusions to be made. Primary associations between the gut microbiome and behavior included *Lactobacillus* and *Ruminoclostridium* spp., while the interaction of *Lactobacillus* with either *Faecalibaculum, Blautia* or *Bifidobacterium* spp. elicited significant secondary associations. The association of *Lactobacillus* spp. with depression has been shown before as exogenous *Lactobacillus* supplementation attenuates symptoms of depression in humans (57) while reduced abundance of *Lactobacillus* and *Bifidobacterium* showed possible clinical associations with MDD (58). Nevertheless, based on the present comprehensive sampling and characterization of the chronic and recurrent-stress induced microbiome in the context of gut microbiota manipulation with prebiotics, probiotics and synbiotics, it can be confirmed that these species, especially *Lactobacillus* spp., are the most significant at predicting stress-induced depressive like behavior and could serve as a biomarker of disease severity with therapeutic implications. As such, *Lactobacillus* spp. have been shown to improve barrier immunity by strengthening the gut epithelium in the context of endotoxin challenge and stimulating the increase in butyrate-producing *Faecalibacterium* and *Anaerotruncus* spp (59). while *Lactobacillus* and *Bifidobacterium* supplementation in aging mice improved barrier function, attenuated peripheral inflammation and simultaneously improved cognitive performance (60).

The impact of the gut microbiota on epithelial integrity can be attributed, in part, to its reprogramming of barrier immunity under homeostatic and challenged conditions. The GALT is the largest secondary lymphoid organ composed of a myriad of immune cells of the innate and adaptive immune systems that are in direct communication with the gut microbiota. The gut microbiota maintains barrier immune homeostasis by modulating the reactivity of resident APCs, facilitating the recruitment of immune cells under times of immune challenge and influencing differentiation of effector T cells (61). These actions alter the barrier and peripheral immune milieu subsequently influencing the inflammatory state of the periphery and the brain. For example, *Lactobacillus* spp. have been shown to influence dendritic cell maturation in mice and consequently bias the effector lymphocyte populations to either Treg (immature dendritic cells) or Th1/Th2 (mature DCs) responses (62) whereas *Bacteriodes fragilis* secretes bacterial polysaccharides (PSA) that alter CD4 T cell reactivity (63). Despite these observations, little is known about the gut microbiota-derived molecular meditators that facilitate this effect or how the microbiota can modulate the interaction of innate immune cells with the naïve effector T cells in the context of chronic and recurrent stress. The comprehensive immunophenotyping with CyTOF in multiple peripheral tissues revealed that variations in the ileal NCR^+^ ILC3 population and correlating upregulation of Treg/Th17 ratio best associated to chronic- and recurrent-stress induced behavioral deficits. The ILCs are a group of innate immune cells expressing interleukin-7 receptor (IL-7Ra/CD127) and are found enriched at mucosal sites in the gut where they contribute to the maintenance of tissue homeostasis and host defense (64). The ILCs form cognate interactions with naïve CD4+ T cells directing their maturation in an activity- and microbiota-dependent manner (65). Murine ILC3 cells, characterized by *Ror*γ*t* and IL-22 expression (66) lack TLRs (67) and are responsive to IL-1β production by macrophages, which stimulates IL-2 production facilitating differentiation of naïve CD4 cells into Tregs. IL-1β production by macrophages is dependent on MYD88- and NOD2-dependent sensing of the microbiota giving the microbiota a key role in determining the functional activity of ILC3 (68). These previously identified mechanisms are in line with the immunological data collected in this study. IL-1β and IL-6 production were primarily associated with the chronic and recurrent behavioral phenotypes in the ileum and PFC. In addition, based on the MARS association of the individual terms within the study covariates, ileal IL-1β production was associated with synbiotic treatment and the stress condition showing ileal IL-1β‘s contribution to recurrent stress-induced behavior. Microbiota-driven IL-1β production by macrophages also promotes GM-CSF (colony-stimulating factor 2 (Csf2)) by ILC3, which in turn stimulates production of retinoic acid and IL-10 driving Treg differentiation (45, 69). Csf2 production by ILC3s additionally promotes the generation of proinflammatory dendritic cells in the spleen (45), while migratory CD103^+^ CD11b^+^ dendritic cells have been implicated in the differentiation of Tregs (70). This putative downstream association was also observed in the current study as migratory splenic CD103^+^ dendritic cells were downregulated due to chronic stress, and uniquely rescued by synbiotic treatment. This also demonstrates how multiple tissues are cooperating to elicit an adaptive response to chronic and recurrent stress following gut microbiota manipulation.

The AHR is another important component of ILC3 activation and the crosstalk of the gut microbiota with barrier immunity (71). Gut microbiota metabolites, especially those derived from tryptophan (i.e. kynurenine) or dietary polyphenols, activate the AHR (72). For example, *Lactobacillus* spp. ferment tryptophan producing indole-3-aldehyde augmenting IL-22 production by ILC3s (30), the stimulator of Treg differentiation. We previously showed that the AHR could be a putative communicator between the gut microbiota and the host in the context of stress-induced psychological impairment due to the variations in tryptophan metabolism (28), and the current study supports this conclusion. From the MARS analysis, ileal *Cyp1a1* transcription, a factor immediately downstream of the AHR, interacted with splenic IL-10 and PFC IL-1β to drive depressive-like behavior. This suggests that AHR signaling in the ileum could be contributing to the neuroinflammatory phenotype in the brain characteristic of chronic and recurrent stress-induced depression. Examining in depth the association of ileal *Cyp1a1* with the study covariates, ileal *Cyp1a1* variations were associated primarily with the behavioral response, and less with the timepoint or treatment conditions suggesting that microbial-AHR-inflammatory signaling could be a fundamental mechanism driving this gut-brain-axis communication.

An important distinction observed in the current study is that ILC3 polarization and subsequent lymphocyte activation is more critical than monocyte or dendritic cell activation for recurrent stress-induced psychological impairment. Previously, groups had postulated that variations in the activation of the innate immune response in MHCII^+^ monocytes (19), CD11c^+^ dendritic cells (10) and/or the infiltration of Ly6C^hi^ monocytes into the brain (20) could be driving sensitivity to recurrent stress. We (28) and other groups (73) also identified that Tregs could drive the sensitivity to chronic and recurrent stress. In this study, comprehensive immunophenotyping of the peripheral and central, innate and adaptive immune responses demonstrated that the microbiome-ILC3-Treg-Th17 axis aligns best with the behavioral phenotypes of chronic and recurrent stress following supplementation with gut microbiota modulating factors (**Figure 6E**). Importantly, we also identified that a synbiotic can indiscriminately attenuate stress-induced alterations in barrier and peripheral immunity and consequently, neuroinflammation. Activated monocytes and dendritic cells remained important for the chronic-stress associated behaviors; however, did not respond to synbiotic treatment nor correspond to the recurrent stress phenotype. As the nature of the machine learning algorithm is correlative, further studies should investigate how removing AHR signaling in Tregs or compromising ILC3 activity causally impacts the behavioral responses to chronic and recurrent stress. Future studies will directly address the question of whether the gut microbiota can alter the recruitment of immune cells into the brain to bring an additional level of understanding to the direct gut-brain-interactions. An important caveat to note is that different mouse strains from different vendors may express correspondingly different immune cell types, mostly due to the presence and activity of the gut microbiota. One group describes how the T:APC cell ratio may determine the functional activity of the immune system, another function of gut microbiota, dependent on strain background and diet (74). Based on this, we can draw these conclusions exclusively for the C57/Bl6 mouse and similar backgrounds on the polyphenol-free diet described in this study.

Epigenetic mechanisms are emerging as an important factor in immunological memory and depression (75, 76). Stress invokes stable epigenetic variations in the innate and adaptive immune cells termed trained adaptive/innate immunity with implications in the development of stress-induced neuropsychiatric disorders (77). In a six-year clinical study of 581 MDD patients, methylation profiles in the blood were found to be highly correlated to disease status with the major themes being immune cell migration and inflammation (78). Importantly, gut microbiota derived metabolites, including the histone deacetylase inhibitor butyrate, can reprogram the cellular epigenome, both proximally and distally (79). Indeed, we previously showed that epigenetic modifications driven by gut microbiota derived metabolites, especially methylation patterns in the IL-6 promoter, promoted resilience to stress-induced depression (80). Specific to immune cell function, ILCs activity is controlled, in part, by their epigenetic landscape. Each ILC subtype contains uniquely regulated enhancer elements enriched with H3K4me3 creating unique motif signatures. Interestingly, H3K4me3 enhancer regions were extensively altered following depletion of the gut microbiota with broad-spectrum antibiotic treatment. This depletion facilitated a shift in the ILC1 and ILC2 populations towards the ROR*γ*t-driven ILC3-like transcriptional profile confirming the dependence of ILC3 on microbial regulation (81). This substantiates the possibility that the synbiotic-specific metabolites may promote resilience to chronic and recurrent stress by invoking epigenetic modifications in the ILC3 cells promoting the differentiation of Tregs over Th17 cells. This, and other specific epigenetic programs should be explored in future studies to understand how cellular imprinting may be contributing to the exaggerated recurrent-stress induced psychiatric phenotypes.

In conclusion, this study describes how the gut-brain-axis can prime the barrier immune response to promote resilience to chronic and recurrent stress associated depressive- and anxiety-like psychological impairment. In particular, synbiotic-specific metabolites can shift ILC3 activity towards a NCR^+^ IL-22 producing phenotype driving an increase in the beneficial Treg/Th17 ratio. Compared to the expression and activation of monocytes, macrophages, dendritic cells and microglia, only the microbiome-ILC3-Th17-Treg axis paralleled the behavioral phenotype in response to chronic and recurrent stress. In addition, these gut microbiota induced variations associated to the release of immune cell recruitment chemokines in the PFC and hippocampus causally linking the different tissues of the gut-brain-axis for chronic and recurrent stress management. Future experiments should explore possible epigenetic mechanisms involving the AHR and associated pathways to causally link specific synbiotic-derived metabolites with immune regulation of stress.

## Data Availability Statement

The original contributions presented in the study are included in the article/**Supplementary Material**. Further inquiries can be directed to the corresponding author.

## Ethics Statement

The animal study was reviewed and approved by Mount Sinai Institutional Animal Care and Use Committee (IACUC).

## Author Contributions

SW conceived, conducted all *in vivo* work, analyzed data and wrote the manuscript. FC assisted SW in all aspects of conducting of the study. TF was instrumental in developing the CUS protocol and assisted in analyzing behavioral data. ME under the supervision of LS developed the MARS algorithm, data representation and interpretation, based on the data provided by SW and GP oversaw all aspects of the study and provided funding. All authors contributed to the article and approved the submitted version.

## Funding

The study was supported by grant number P50 AT008661-01 and U19 AT010835 from the NCCIH and the ODS.

## Conflict of Interest

The authors declare that the research was conducted in the absence of any commercial or financial relationships that could be construed as a potential conflict of interest.
