# Supplementary material for: Chronic Stress-Induced Depression and Anxiety Priming Modulated by Gut-Brain-Axis Immunity
Source: Front Immunol. 2021 Jun 24;12:670500. doi: 10.3389/fimmu.2021.670500 (PMC8264434; doi:10.3389/fimmu.2021.670500)
Supplement: Supplementary file 1 [file DataSheet_1.pdf]

Supplementary Information

Figures

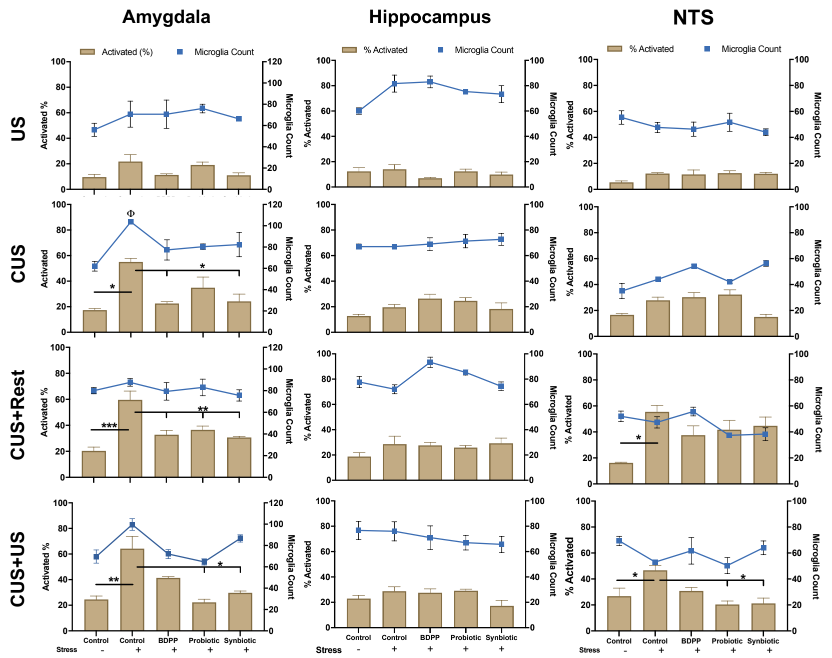

Supplementary Figure 1: Microglia activation in limbic brain regions during the CUS protocol.

Microglia activation was assessed as the percentage of surface area covered by the activation marker CD68 compared to the total area stained with Iba1 (brown columns, left axis). Microglia count in each frame is also displayed (blue line, right axis). Measures were taken at all timepoints including US, CUS, CUS+Rest and CUS+US. Significance (*n* = 3, +/- SEM) was determined with a one-way ANOVA and Tukey's post-hoc analyses and marked as *p < 0.05, **p < 0.01, ***p < 0.0001.

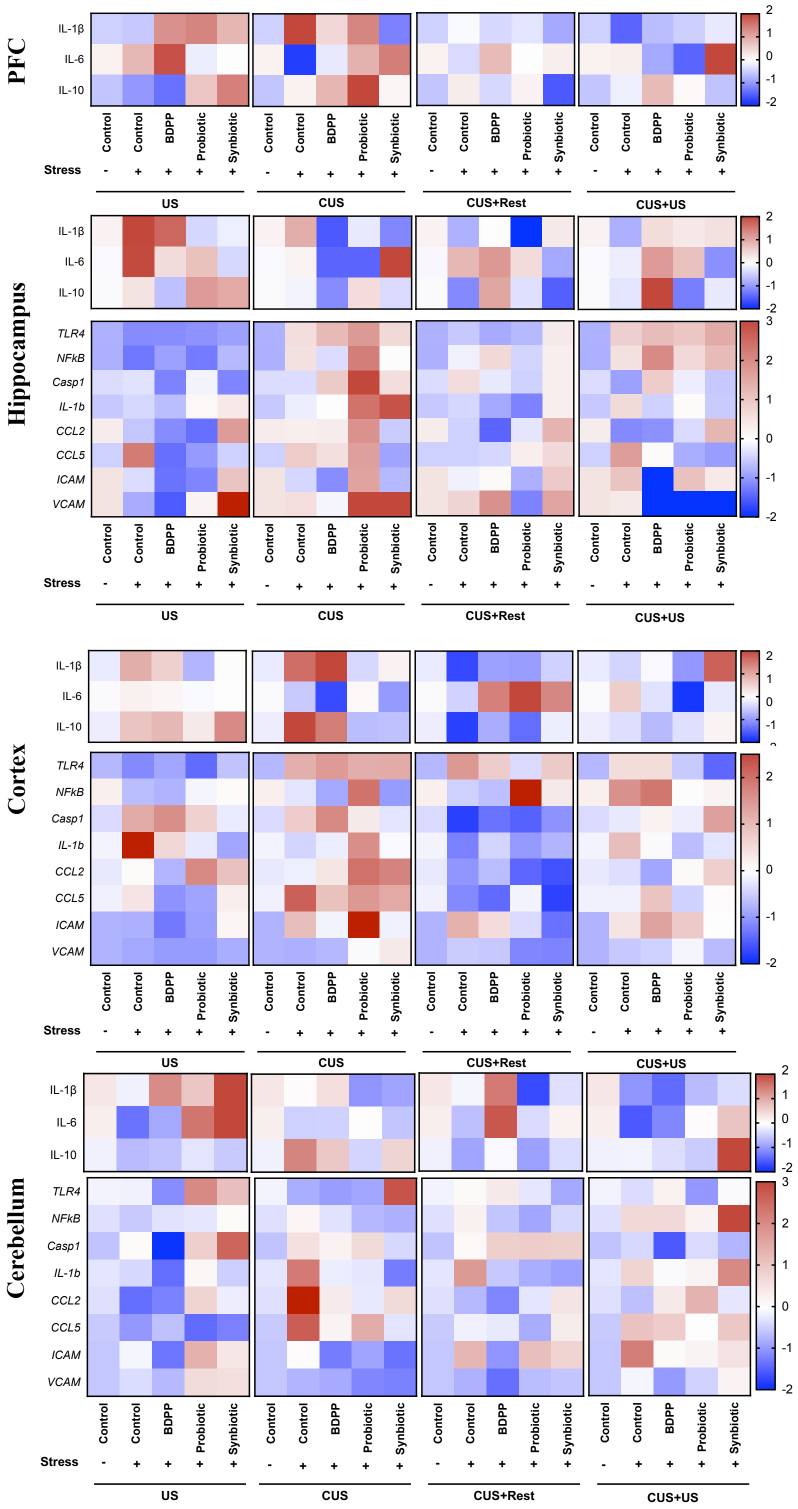

Supplementary Figure 2: Gut microbiota altering substances modulate gene and cytokine expression of immunological factors during chronic and recurrent stress.

Gene and cytokine expression in the prefrontal cortex (PFC), hippocampus, cortex and cerebellum is outlined as the z-score of relative expression normalized across all timepoints for a

particular gene or cytokine. Note that each instance is a ratio of stressed vs. non-stressed for each of the respective treatment group. For each group, n = 6 individuals are shown.

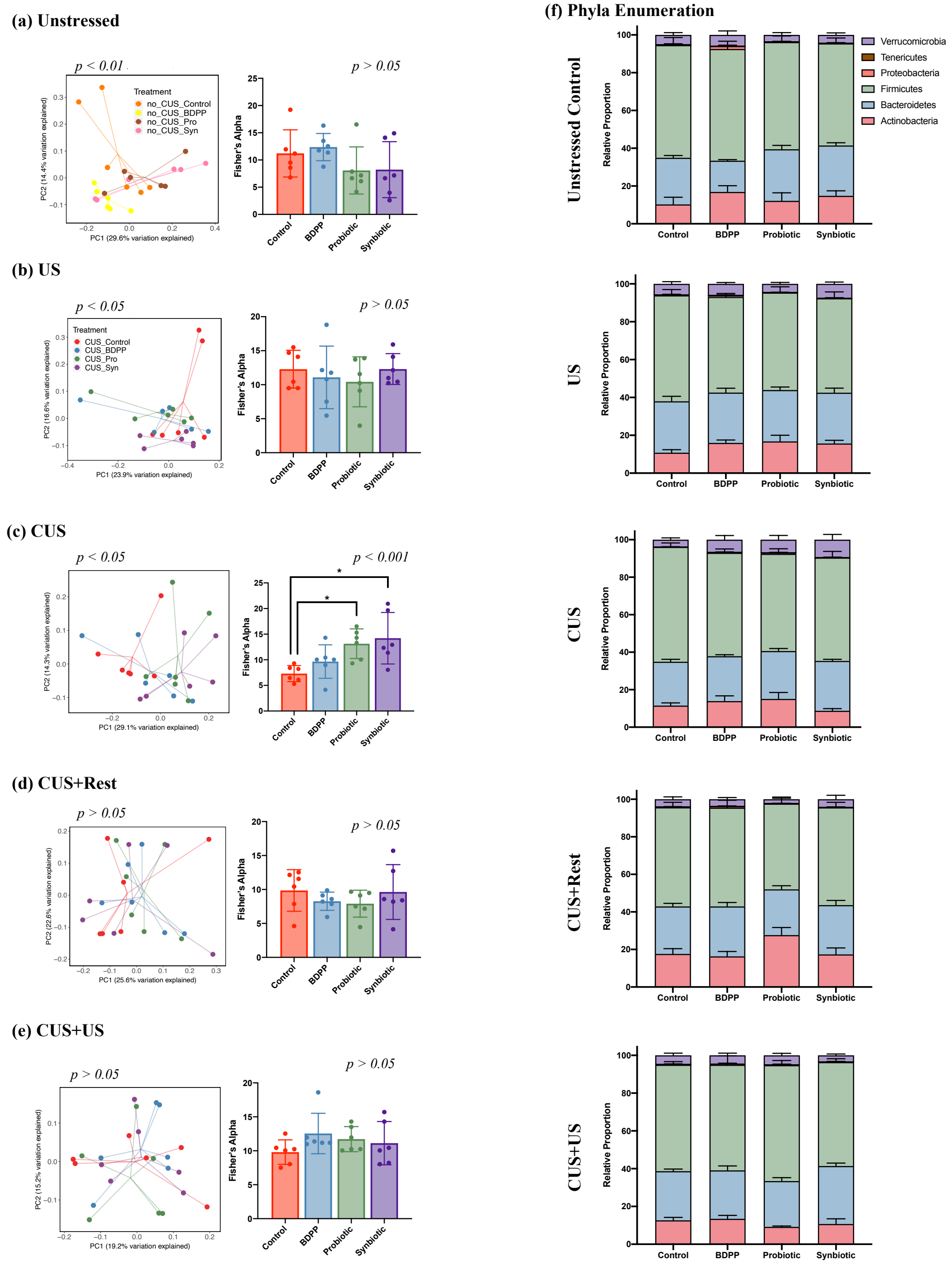

Supplementary Figure 3: Gut microbiome variations in response the CUS protocol. Alpha diversity (Fisher's alpha) and beta diversity (unweighted UniFrac PCoA plots) show the variations in the gut microbiome with respect to the CUS protocol within a treatment group for **(a)** CUS control vs. stressed, **(b)** Control, **(c)** BDPP, **(d)** Probiotic and **(e)** Synbiotic. **(f)** Enumeration of the main phyla across treatment at the respective timepoints is also shown. Each group contains n = 6 individuals +/- SEM with significance determined with one-way ANOVA *p<0.05 and **p<0.01.

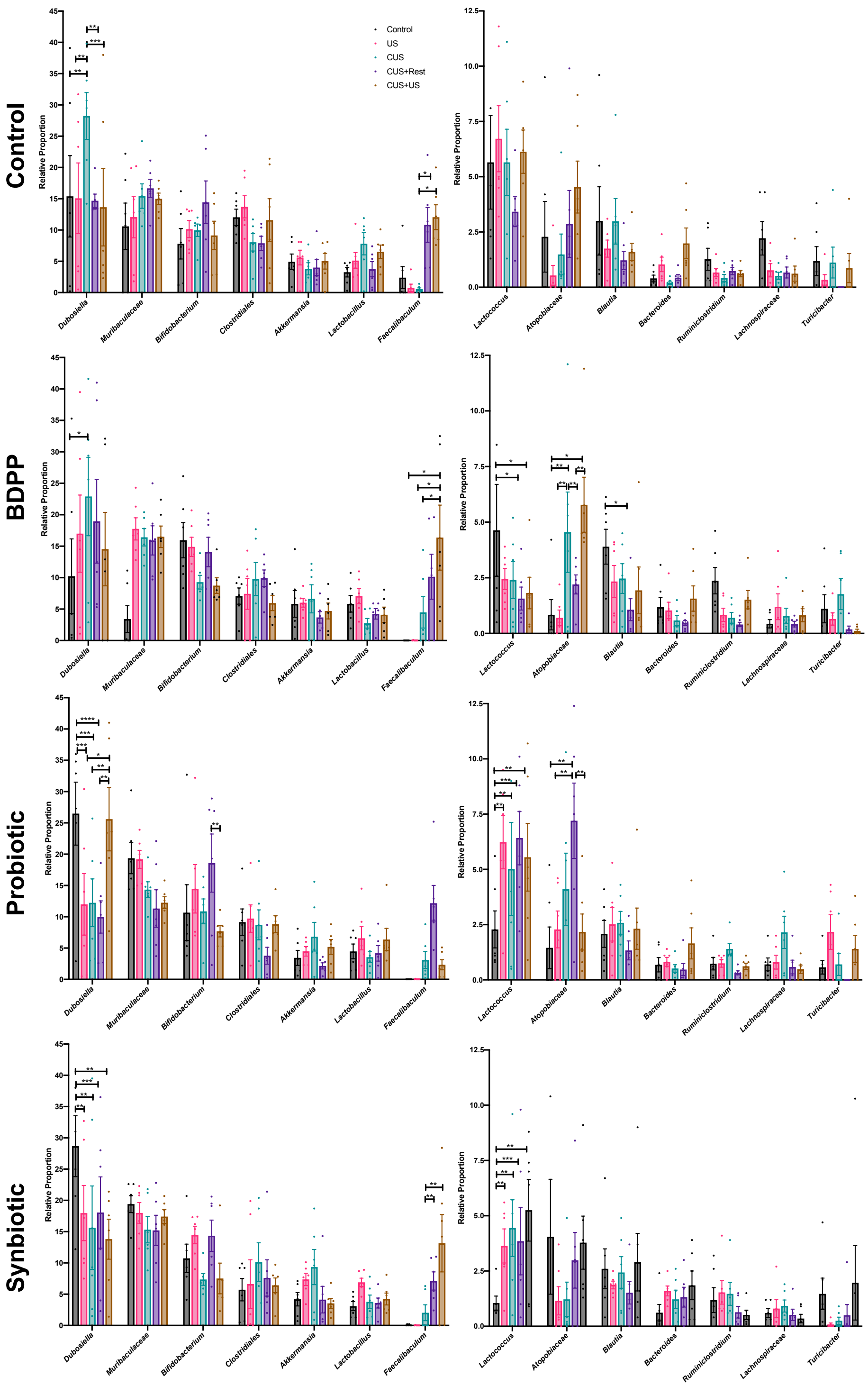

Supplementary Figure 4: Gut microbiome genera variations with respect to time point during chronic and recurrent stress. 16S *V4* sequencing of gut microbiome genera of stressed mice only during the CUS protocol are shown with variations with respect to timepoint during the CUS protocol for the respective treatments. Each group represents *n* = 6 individuals +/- SEM with significance determined with a one-way ANOVA and Tukey's post-hoc analysis where *p<0.05, **p<0.01 and ***p<0.001.

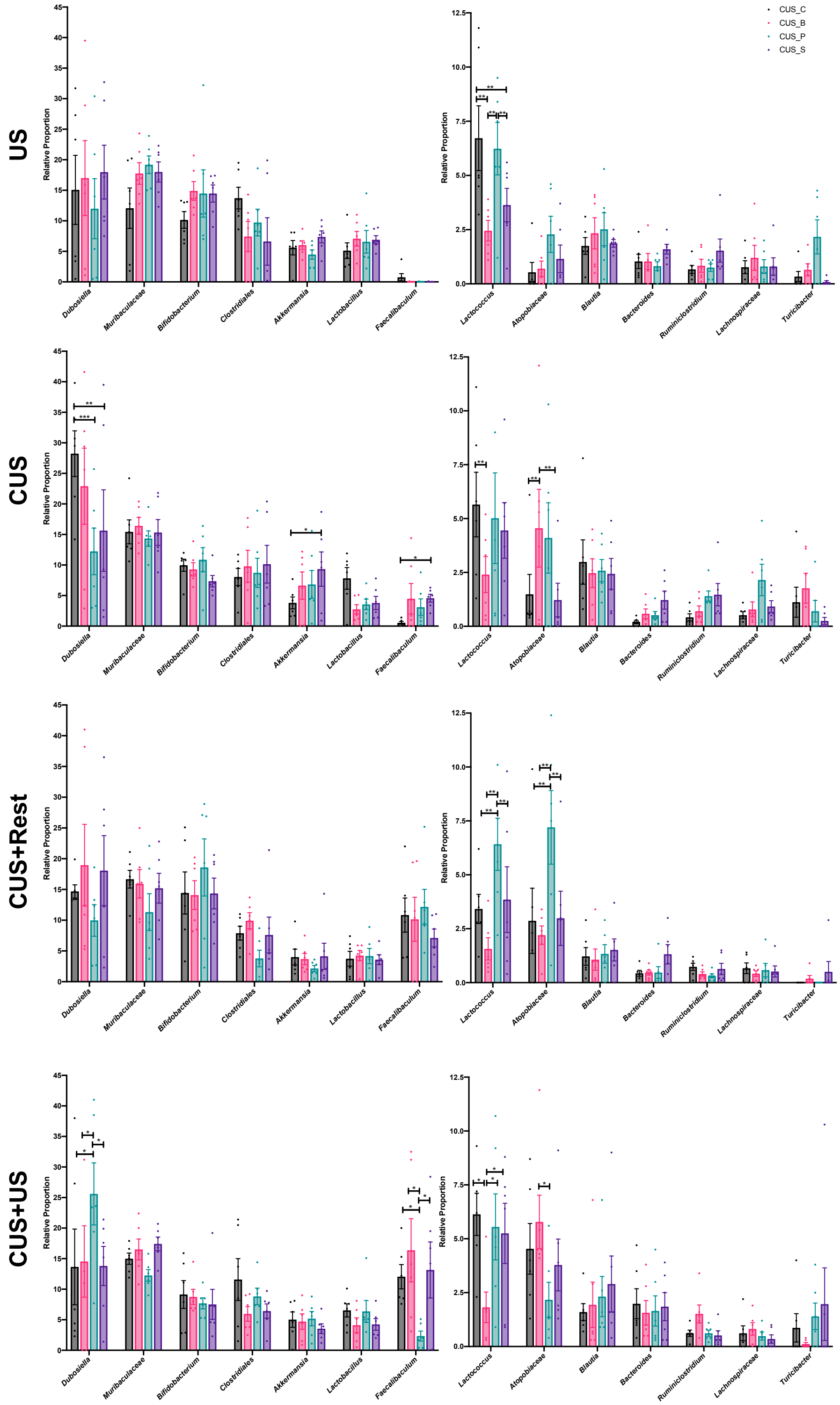

Supplementary Figure 5: Gut microbiome genera variations with respect to treatment during chronic and recurrent stress. 16S *V4* sequencing of gut microbiome genera of stressed mice only during the CUS protocol are shown with variations with respect to treatment. Each group represents *n* = 6 individuals +/- SEM with significance determined with a one-way ANOVA and Tukey's post-hoc analysis where *p<0.05, **p<0.01 and ***p<0.001.

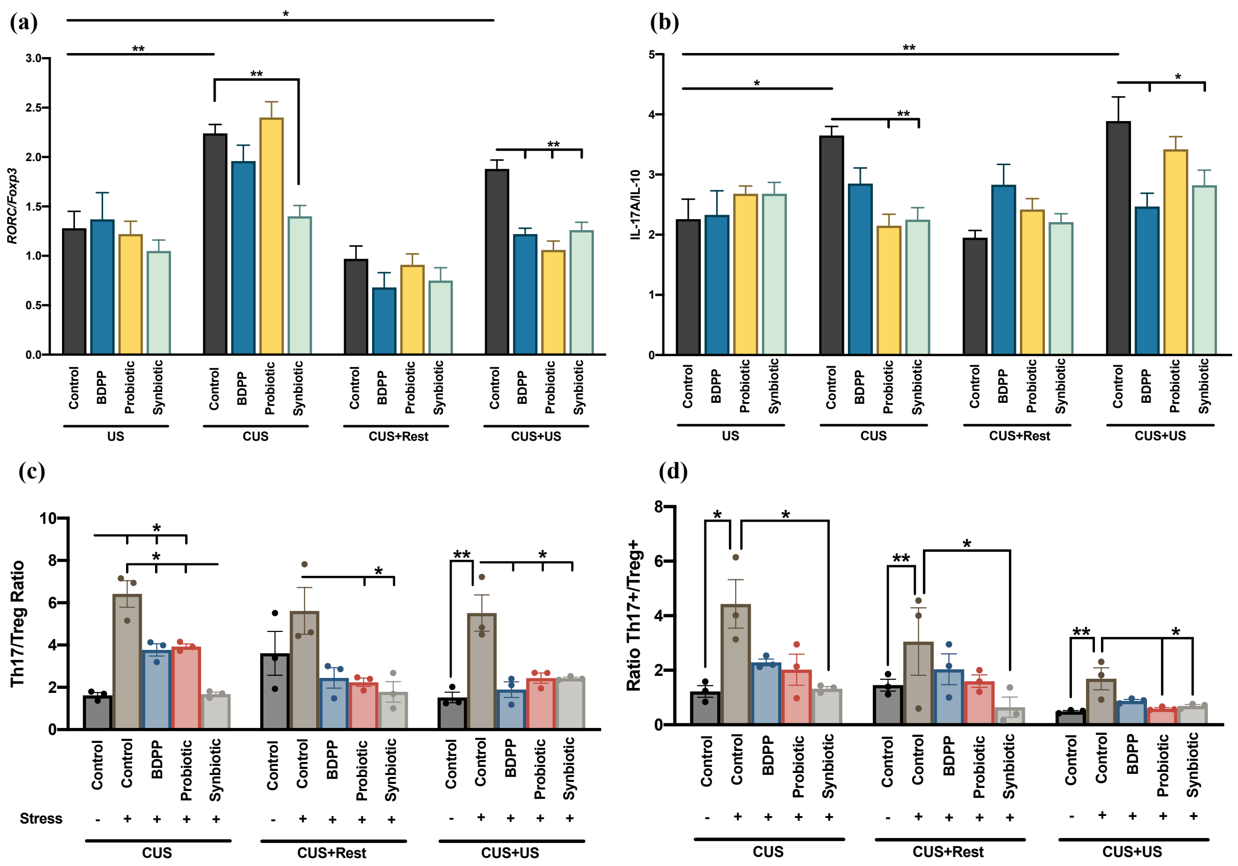

Supplementary Figure 6: Stress and synbiotic induced variations on the ratio of Th17 to Treg and their activated states. The ratios of Th17 to Treg cells was determined with **(a)** the ratio of transcription factors *Rorγt* to *Foxp3,* **(b)** ratio of cytokines IL-17A to IL-10, **(c)** the ratio of unactivated Th17/Treg cells and **(d)** ratio of "activated" Th17 (IL-17A^{+}ICOS^{+}) and Treg (CTLA4^{+}). For the gene and cytokine expression, n = 6 independent samples +/- SEM were used, while n = 3 individuals for the cellular data, while statistics were calculated with one-way ANOVAs in all cases with Tukey's post-hoc analysis *p<0.05, **p<0.01.

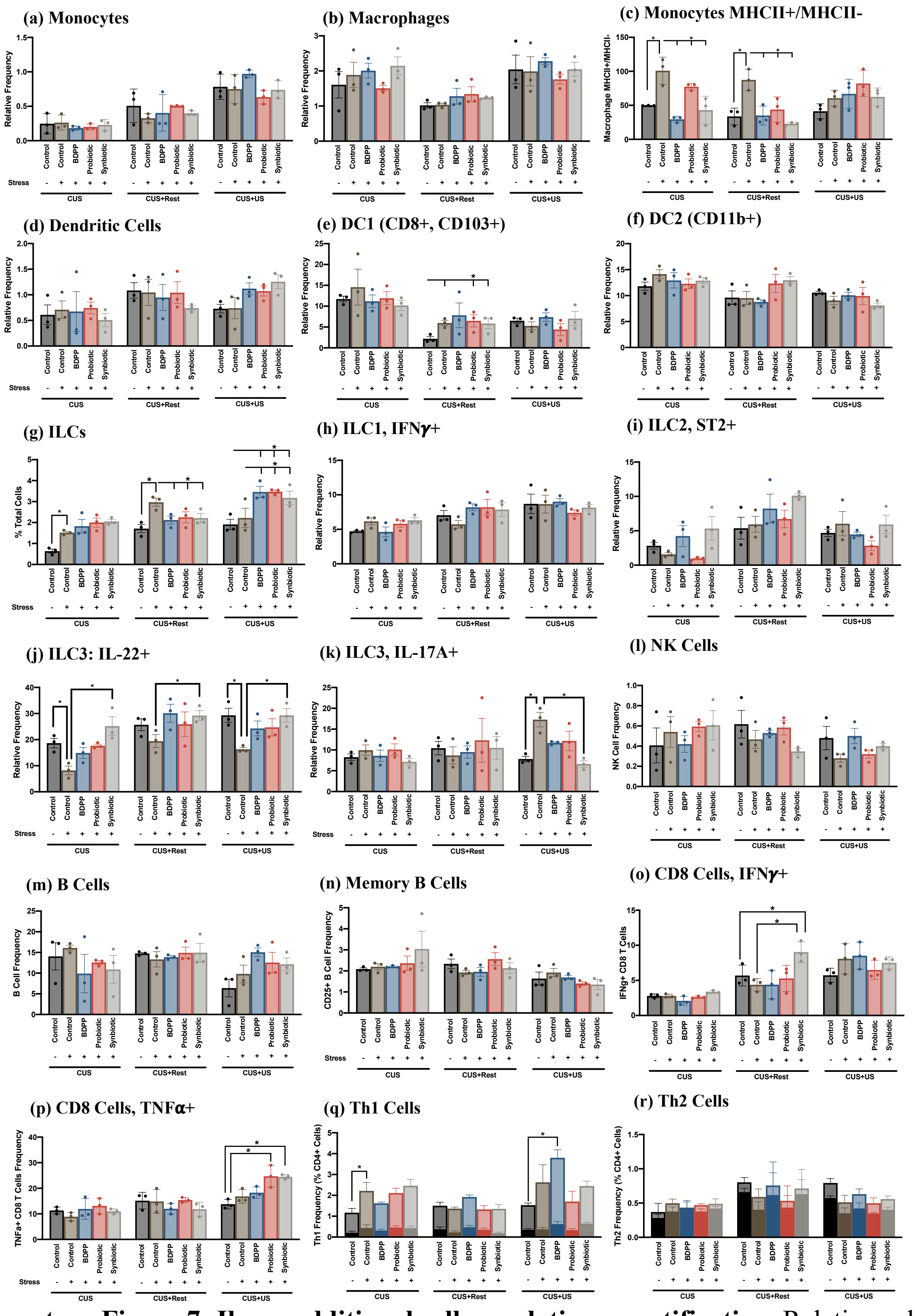

Supplementary Figure 7: Ileum additional cell population quantification. Relative cellular expression (as indicated on y-axis) or activation for various cell populations and their activation states are shown for **(a)** monocytes relative to total cell populations, **(b)** macrophages relative to total cell populations, **(c)** ratio of MHCII$^+$ to MHCII$^-$ cells, **(d)** dendritic cells (DC) relative to total cell populations, **(e)** DC1 and **(f)** DC2 relative to total DC cell population, **(g)** innate

lymphoid cells (ILCs) relative to total cell populations, **(h)** ILC1, **(i)** ILC2, **(j)** ILC3 IL-22^{+} and **(k)** ILC3 IL-17A^{+} relative to ILC populations, **(l)** NK cells relative to total cell populations, **(m)** B cells and **(n)** memory B cells relative total cell populations, **(o)** CD8 IFNɣα cells and **(p)** CD8 TNFα cells relative to CD8 cell populations and **(q)** Th1 and **(r)** Th2 cells relative to total cell populations in their naïve and activated states (solid superimposed bars). In all cases, *n* = 3 individuals +/- SEM are shown and significance indicated with a one-way ANOVA with Tukey's post-hoc analysis with *p<0.05, **p<0.01.

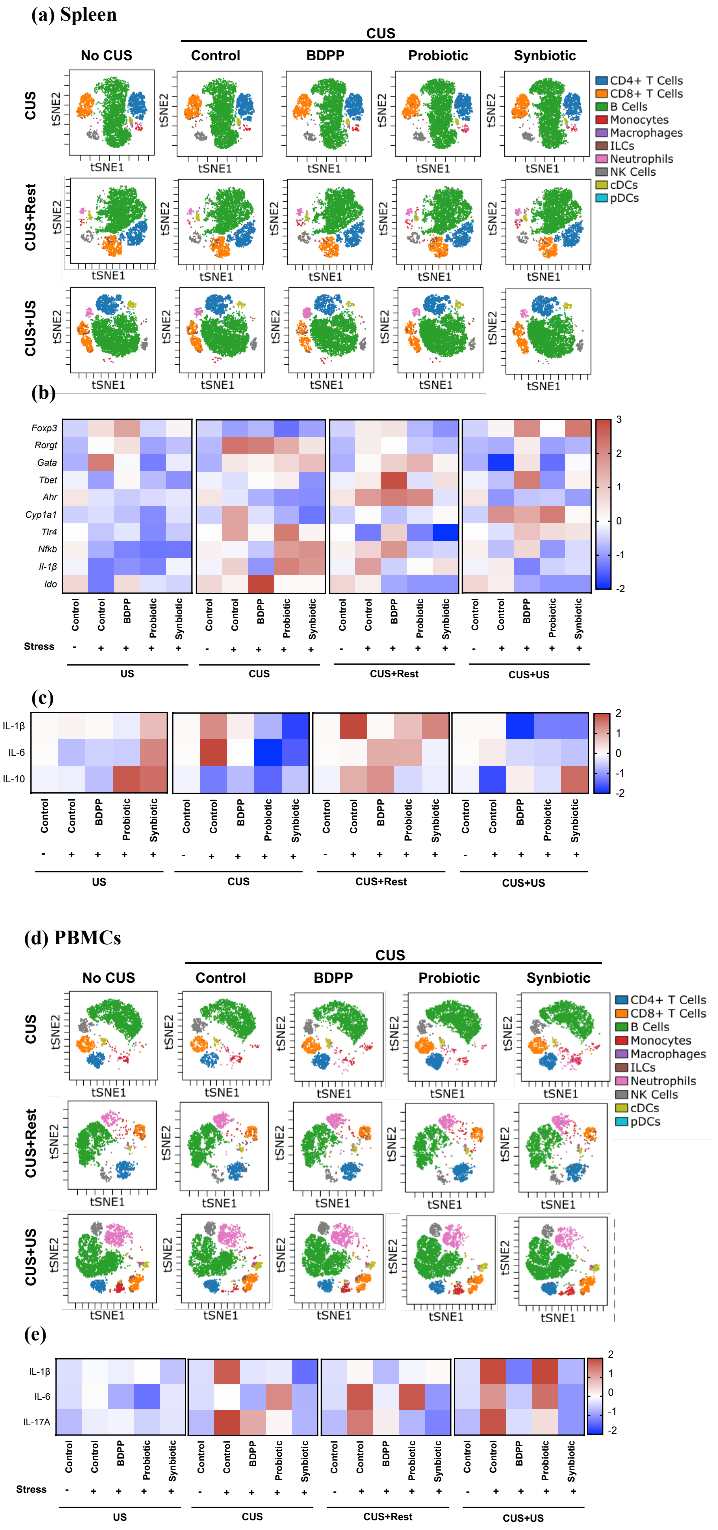

Supplementary Figure 8: Immune cell distribution in spleen and PBMCs. Major cell populations determined with CyTOF immunophenotyping in the **(a)** spleen and quantification of **(b)** genes and **(c)** cytokines in the spleen are shown as heatmaps with each instance being the ratio of stressed vs. non-stressed group within each treatment, with the z-score taken across time for each gene. Major cell populations from **(d)** PBMCs are represented in viSNE plots while

proinflammatory cytokine expression in the serum is shown as a heatmap in **(e)**. For the cell frequencies, *n* = 3 independent samples +/- SEM are shown while for the gene and cytokine determination, *n* = 6 +/- SEM is shown.

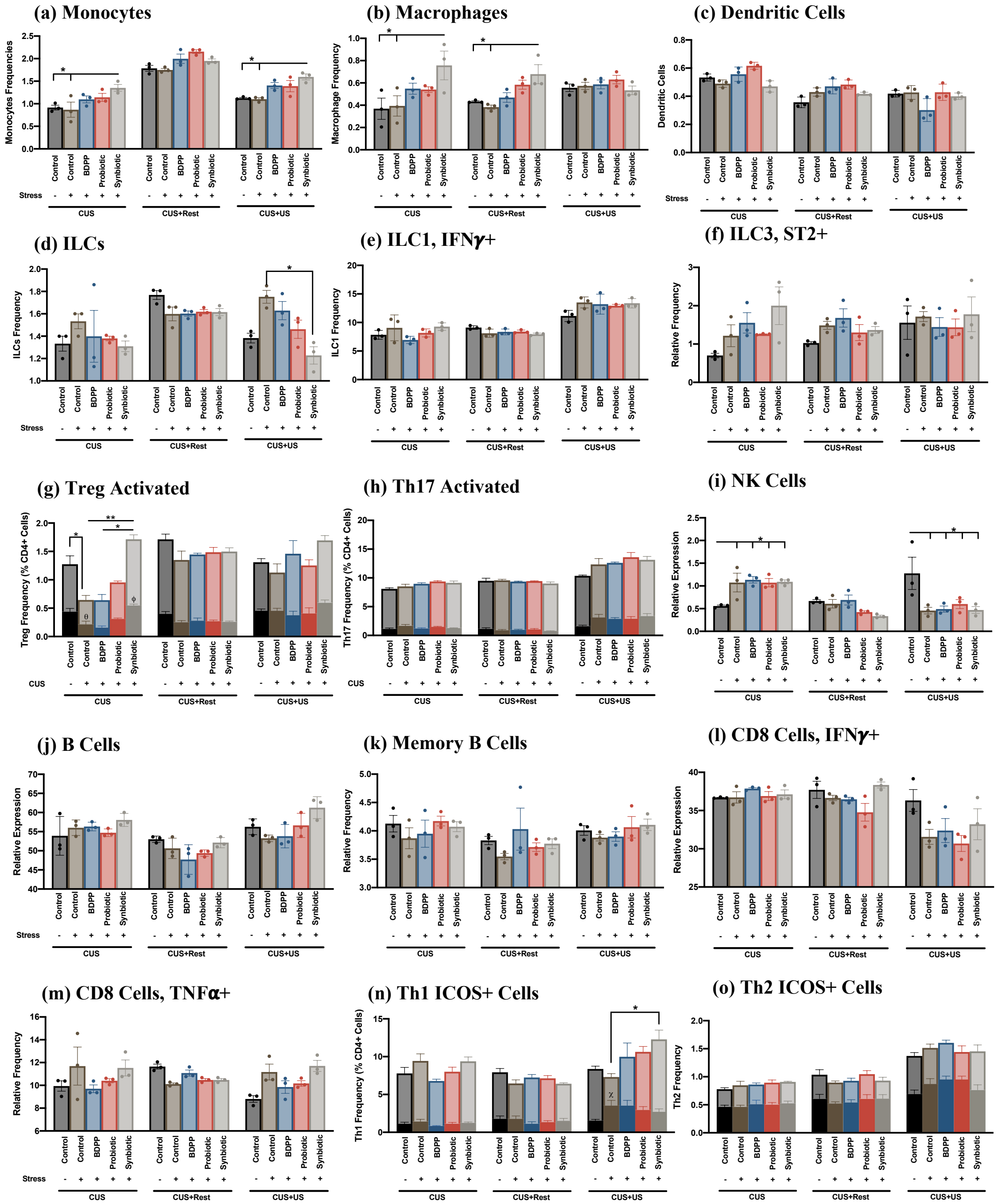

Supplementary Figure 9: Spleen additional cell population quantification. Relative cellular expression (as indicated on y-axis) or activation for various cell populations and their activation states are shown for **(a)** monocytes relative to total cell populations, **(b)** macrophages relative to total cell populations, **(c)** dendritic cells (DC) relative to total cell populations, **(d)** innate lymphoid cells (ILCs) relative to total cell populations, **(e)** ILC1 and **(f)** ILC3 relative to total

cell populations, **(g)** regulatory T cells (Tregs) and **(h)** T helper (Th)17 cells, both with an inset of activated cell populations (Treg, $CTLA4^+$ and Th17, $IL17A^+ICOS^+$) relative to total CD4+ cells, **(i)** NK cells relative to total cell populations, **(j)** B cells and **(k)** memory B cells relative total cell populations, **(l)** CD8 IFNɣ cells and **(m)** CD8 TNFα cells relative to total CD8 cell populations and **(n)** Th1 and **(o)** Th2 cells relative to total CD4+ cell populations in their naïve and activated states (solid superimposed bars). In all cases, $n = 3$ individuals +/- SEM are shown and significance indicated with a one-way ANOVA with Tukey's post-hoc analysis with $*p<0.05$, $**p<0.01$.

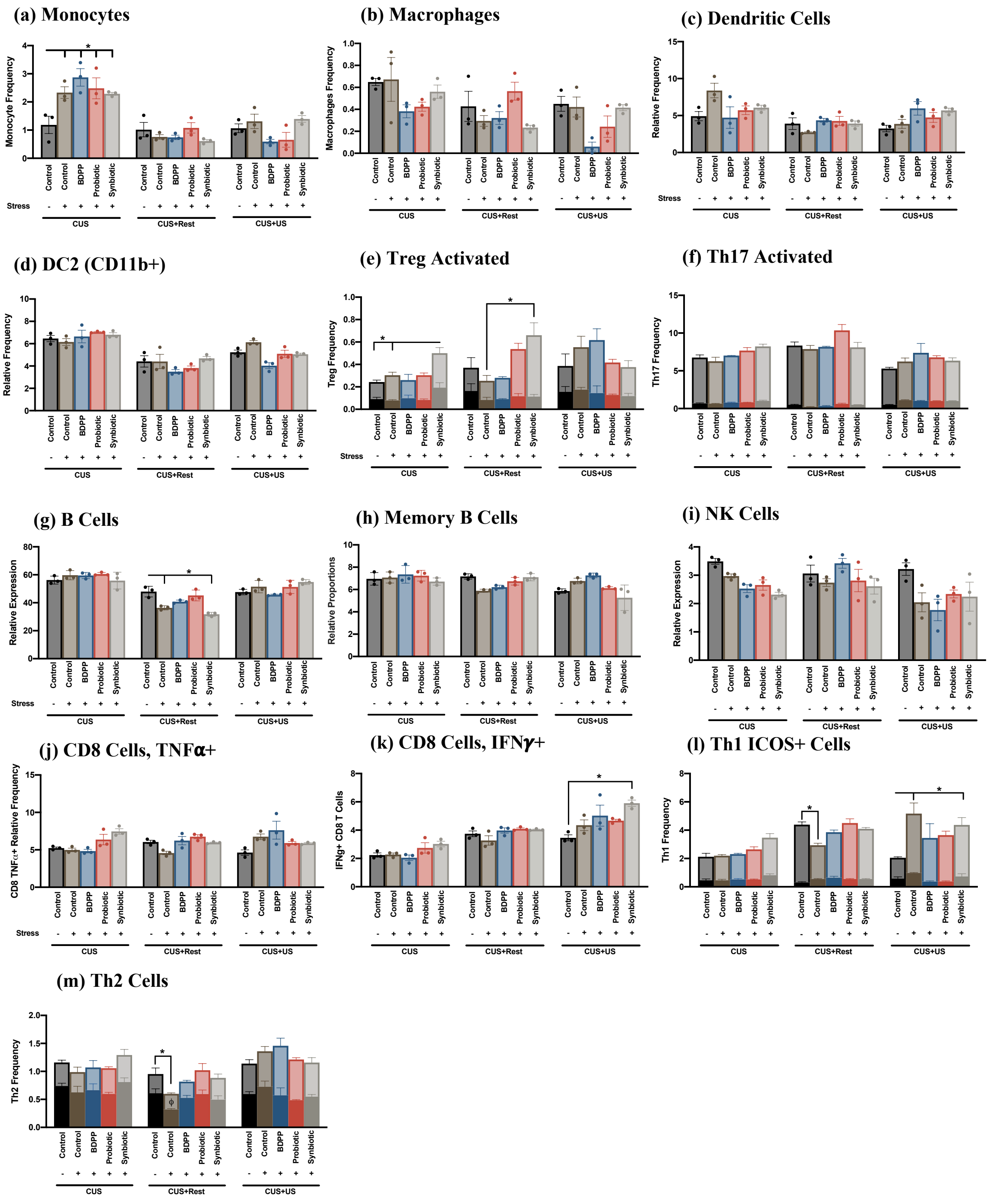

Supplementary Figure 10: PBMC additional cell population quantification. Relative cellular expression (as indicated on y-axis) or activation for various cell populations and their activation states are shown for **(a)** monocytes relative to total cell populations, **(b)** macrophages relative to total cell populations, **(c)** dendritic cells (DC) relative to total cell populations, **(d)** DC2 relative to total DC cell populations, **(e)** regulatory T cells (Tregs) and **(f)** T helper (Th)17 cells, both with an inset of activated cell populations (Treg, $CTLA4^{+}$ and Th17, $IL17A^{+}ICOS^{+}$) relative to total CD4+ cells, **(g)** B cells and **(h)** memory B cells relative total cell populations, **(i)** NK cells relative to total cell populations, **(j)** CD8 TNFα cells and **(k)** CD8 IFNγ cells relative to total

CD8 cell populations and **(l)** Th1 and **(m)** Th2 cells relative to total CD4+ cell populations in their naïve and activated states (solid superimposed bars). In all cases, n = 3 individuals +/- SEM are shown and significance indicated with a one-way ANOVA with Tukey's post-hoc analysis with *p<0.05, **p<0.01.

Supplementary Table 1: Polyphenol-free Diet Composition

The polyphenol-free diet was custom modified from Research Diets Ain-93G diet (New Brunswick, NJ, USA)

Class Description	Ingredient	Grams
Protein	Casein, Lactic, 30 Mesh	200 g
Protein	Cystine, L	3 g
Carbohydrate	Starch, Corn	397 g
Carbohydrate	Maltodextrin 10	132 g
Carbohydrate	Sucrose, Fine Granulated	100 g
Fiber	Cellulose, BW200	50 g
Fat	Corn Oil	70 g
Mineral	S10022G	35 g
Vitamin	V10037	10 g
Vitamin	Choline Bitartrate	2.5 g
		**Total: 1000 g**

Caloric Information	
Protein	20.3 % kcal
Fat	15.8 % kcal
Carbohydrate	63.9 % kcal
Energy Density	4.00 kcal/g

Supplementary Table 2: Chronic Unpredictable Stress Protocol

Day	Stress 1	Stress 2
**Week 1**		
Monday	Crowding, 60 min	Cage Tilt, 45°, 12h (7PM - 7AM)
Tuesday	Cold Swim (4°C, 5min)	Lights ON 12h, (6PM - 6AM)
Wednesday	Cage Shaking, 20 min	Food deprivation, 12h (7PM - 7AM)
Thursday	Wet Bedding, 6hr (10am-4pm)	Forced swim, 5min, 4°C
Friday	Restraint Stress, 1hr	Cage Tilt, 45°, 12h (7PM – 7AM)
Saturday	Fox odor, 10h (8AM – 6PM)	Lights ON 12h, (6PM - 6AM)
Sunday	Water deprivation, 10h (10AM-6PM)	Cold exposure (4C, 1hr)
**Week 2**		
Monday	Forced swim, 5min, 4°C	No Bedding (12h, 6PM - 6AM)
Tuesday	Cage Shaking, 20 min	Fox Odor (12h, 8PM - 8AM)
Wednesday	Restraint Stress, 1hr	Water Deprivation (8h, 12AM - 8AM)
Thursday	Forced swim, 5min, 4°C	Cold Exposure (20min, 4C)
Friday	Wet Bedding, 10h (10AM – 8PM)	No Bedding, 12 h (8PM-8AM)
Saturday	Cage Tilt, 45°, 12h (7AM-7PM)	Fox odor, 10h (10PM – 8AM)
Sunday	Cage Shaking, 20 min	Cage Tilt, 45°, 12h (7PM – 7AM)
**Week 3**		
Monday	Cold Swim (4°C, 5min)	Food&Water Dep., 12h, (8PM-8AM)
Tuesday	Cage Shaking, 20 min	Lights ON 12h, (6PM - 6AM)
Wednesday	Crowding, 60 min	Cage Tilt, 45° , 12h (7PM – 7AM)
Thursday	Wet Bedding, 8h (10AM-6PM)	Restraint Stress, 1hr
Friday	Food Deprivation, 12h (8AM-8PM)	No Bedding, 12 h (8PM-8AM)

Saturday	Fox odor, 8h (10AM – 6PM)	Lights ON 12h, (6PM - 6AM)
Sunday	Restraint Stress, 1hr	Wet Bedding, 8h (10AM-6PM)
**Week 4**		
Monday	Fox odor, 10h (10AM – 8PM)	Cage Tilt, 45°, 8h (12PM – 8AM)
Tuesday	Cold Swim (4°C, 5min)	Food&Water Dep., 12h, (8PM-8AM)
Wednesday	Cage Shaking, 20 min	Wet Bedding, 10h (10PM-8AM)
Thursday	Crowding, 60 min	Restraint Stress, 1hr
Friday	Cold exposure (4C, 1hr)	Wet Bedding, 10h (10PM-8AM)
Saturday	No Bedding, 10h (8AM-6PM)	Cage Tilt, 45°, 8h (12PM – 8AM)
Sunday	Crowding, 60 min	Fox odor, 10h (10AM – 8PM)

**Subthreshold US**		
Monday	Fox odor, 10h (10AM – 8PM)	Cage Tilt, 45°, 8h (12PM – 8AM)
Tuesday	Cold Swim (4°C, 5min)	Food&Water Dep., 12h, (8PM-8AM)
Wednesday	Cage Shaking, 20 min	Wet Bedding, 10h (10PM-8AM)
Thursday	Crowding, 60 min	Restraint Stress, 1hr
Friday	Cold exposure (4C, 1hr)	Wet Bedding, 10h (10PM-8AM)
Saturday	No Bedding, 10h (8AM-6PM)	Cage Tilt, 45°, 8h (12PM – 8AM)
Sunday	Crowding, 60 min	Fox odor, 10h (10AM – 8PM)

Supplementary Table 3: Mouse Primer Sequences

Gene	Accession Number	Sequence (5' – 3')	Product Length (bp)
TLR2	NM_011905.3	F: CCGAAACCTCAGACAAAGCGTCA<br>R: TCACACACCCCAGAAGCATCACAT	144
TLR4	NM_021297.3	F: GCAAAGTCCCTGATGACATTCCTT<br>R: CCACAGCCACCAGATTCTCTAAA	267
IL-6	NM_001314054.1	F: CACAAGTCCGGAGACCAGAC<br>R: CAGAATTGCCCATTGCACAAC	142
IL-1β	NM_008361.4	F: TTCAGGCAGGCAGTATCACTC<br>R: CCACGGGAAAGACACAGGTAG	69
Nlrp3	NM_145827.4	F: AGAAGAGACCACGGCAGAA<br>R: CCTTGGACCAGGTTCAGTGT	102
Casp1	NM_009807.2	F: CACATTTCCAGGACTGACTGG<br>R: AGACGTGTACGAGTGGTTGT	120
NFκB	NM_021151352.2	F: CTGGTGGACACATACAGGAAGAC<br>R: ATAGGCACTGTCTTCTTTCACCTC	195
ICAM	NM_010493.3	F: GTTTAAAACCAGACCCTGGAACT<br>R: CGTCTGCAGGTCATCTTAGGAG	152
VCAM	NM_011693.3	F: CCGGCATATACGAGTGTGAAT<br>R: ATGGCAGGTATTACCAAGGAAGAT	151
IL-10	NM_010548.2	F: GCTCTTACTGACTGGCATGAG<br>R: CGCAGCTCTAGGAGCATGTG	105
CCL5	NM_013653.3	F: AGATCTCTGCAGCTGCCCTCA<br>R: GGAGCACTTGCTGCTGGTGTAG	170
CCL2	NM_011333.3	F: GCATCCACGTGTTGGCTCA<br>R: CTCCAGCCTACTCATTGGGATCA	95
AHR	NM_001314027.1	F: ACCAGAACTGTGAGGGTTGG<br>R: TCTGAGGTGCCTGAACTCCT	155
IDO	NM_145949.2	F: CCTCATCCCTCCTTCCTTTC<br>R: GGAGCAATTGCCTGGTATGT	219
Foxp3	NM_001199348.1	F: TTGGCCAGCGCCATCTT<br>R: TGCCTCCTCCAGAGAGAAGTG	100
Tbet	XM_021177762.1	**F: AGCAAGGACGGCGAATGTT**<br>**R: GGGTGGACATATAAGCGGTTC**	187
Gata3	XM_029547696.1	**F: CGGCCATTCGTACATGGAA**<br>**R: GGATACCTCTGCACCGTAGC**	135
RORC	NM_001293734.1	**F: GTGGAGTTTGCCAAGCGGCTTT**<br>**R: CCTGCACATTCTGACTAGGACG**	108
IL-17A	NM_010552.3	F: AAGGCAGCAGCGATCATCC<br>R: GGAACGGTTGAGGTAGTCTGAG	150
Cyp1a1	NM_001136059.2	F: GGGTTTGACACAGTCACAACT	197

		R: GGGACGAAGGATGAATGCCG	
IL23R	NM_144548.1	**GTCCACCAAACTTCCCAGACAG**	110
		**CCTGAAGCAGGATGTCCTCTGA**	
GAPDH	NM_001289726.1	F: ATGTGTCCGTCGTGGATCTGAC	132
		R: AGACAACCTGGTCCTCAGTGTAG	

Supplementary Table 4: Target Antibodies, clone and conjugate for CyTOF Experiments
Tags marked with an asterisk (*) were used in calculating the viSNE analysis

Target	Clone	Source	Metal Conjugate
CD45	30-F11	Biolegend	112Cd, 114Cd, 116Cd
MHCI(H-2)*	M1/42	Biolegend	112Cd, 114Cd, 116Cd
Ly-6G*	1A8	Fluidigm	141 Pr
CD11c*	N418	Biolegend	142 Nd
TCRb*	H57-597	Fluidigm	143 Nd
CD115*	AFS98	Fluidigm	144 Nd
F4/80*	BM8	Fluidigm	146 Nd
CD11b*	M1/70	Biolegend	148 Nd
CD19*	6D5	Biolegend	149 Sm
CD25*	3C7	Biolegend	151 Eu
CD3e*	145-2C11	Fluidigm	152 Sm
CD16/32	93	Fluidigm	153 Eu
CTLA4	UC10-4B9	Fluidigm	154 Sm
ST2*	DIH9	Biolegend	155 Gd
Foxp3	FJK-16s	Fluidigm	158 Gd
RORgt	B2D	Fluidigm	159 Tb
B220*	RA3-6B2	Fluidigm	160 Gd
CD103*	2E7'	Biolegend	161 Dy
TNFα	MP6-T22	Fluidigm	162 Dy
IL-23R*	polyclonal	Novus Bio	163 Dy
Sca-1	D7	Fluidigm	164 Dy
IFNγ	XMG1.2	Fluidigm	165 Ho
CD117*	2B8	Biolegend	166 Er
NKp46*	29A1.4	Fluidigm	167 Er
CD8*	53-6.7	Biolegend	168 Er
IL22*	NBP2-11699	Novus Bio	169 Tm
NK1.1*	PK136	Fluidigm	170 Er
CD4*	RM4-5	Biolegend	172 Yb
IL-17A	TC11-18H10.1	Fluidigm	174 Yb
CD127*	A7R34	Biolegend	175 Lu
ICOS*	7E.17G9	Fluidigm	176 Yb
MHCII (I-A/I-E)	M5.114.15.2	Fluidigm	209 Bi

Supplementary Table 4: Fold change of relative gene expression of inflammatory markers in the prefrontal cortex. Detailed gene expression analysis of data presented in figure 2b. Each value is represented as the fold change of the relative gene expression (relative to *gapdh*) from stressed vs. unstressed controls at the same timepoint. Each value is the mean of n = 6 independent samples +/- SEM.

		US	CUS	CUS+Rest	CUS+US
TLR4	Control	0.79 ± 0.22	1.66 ± 0.20	1.22 ± 0.21	1.50 ± 0.18
	BDPP	1.32 ± 0.25	1.00 ± 0.29	1.16 ± 0,15	0.95 ± 0.18
	Probiotic	0.72 ± 0.44	1.81 ± 0.13	0.96 ± 0.21	1.27 ± 0.26
	Synbiotic	0.89 ± 0.20	1.11 ± 0.21	1.13 ± 0.19	0.98 ± 0.24
Nfkb	Control	0.97 ± 0.21	1.01 ± 0.14	0.91 ± 0.23	0.94 ± 0.24
	BDPP	1.25 ± 0.21	0.73 ± 0.23	1.03 ± 0.13	0.78 ± 0.11
	Probiotic	1.15 ± 0.24	1.10 ± 0.19	0.95 ± 0.16	1.05 ± 0.25
	Synbiotic	1.24 ± 0.17	0.93 ± 0.15	1.06 ± 0.17	0.88 ± 0.21
Casp1	Control	1.30 ± 0.20	1.45 ± 0.30	0.77 ± 0.22	1.62 ± 0.30
	BDPP	0.99 ± 0.31	1.16 ± 0.24	1.26 ± 0.30	0.73 ± 0.34
	Probiotic	1.14 ± 0.26	1.69 ± 0.23	0.88 ± 0.30	1.04 ± 0.32
	Synbiotic	1.21 ± 0.30	1.13 ± 0.21	0.97 ± 0.36	1.25 ± 0.27
Il1b	Control	0.99 ± 0.37	1.91 ± 0.41	1.00 ± 0.24	1.60 ± 0.28
	BDPP	1.41 ± 0.39	1.57 ± 0.25	0.89 ± 0.23	0.80 ± 0.26
	Probiotic	1.05 ± 0.44	1.81 ± 0.39	1.01 ± 0.22	1.19 ± 0.09
	Synbiotic	1.11 ± 0.30	1.57 ± 0.30	1.10 ± 0.23	0.75 ± 0.42
CCL2	Control	0.97 ± 0.32	2.13 ± 0.23	1.54 ± 0.15	3.50 ± 0.16
	BDPP	1.48 ± 0.09	1.68 ± 0.23	1.17 ± 0.19	1.95 ± 0.18
	Probiotic	1.33 ± 0.44	3.54 ± 0.32	1.58 ± 0.22	0.78 ± 0.40
	Synbiotic	1.32 ± 0.26	1.87 ± 0.26	1.57 ± 0.17	1.00 ± 0.13
CCL5	Control	0.83 ± 0.06	2.21 ± 0.16	1.09 ± 0.14	1.68 ± 0.15
	BDPP	0.65 ± 0.05	1.25 ± 0.17	1.28 ± 0.16	1.59 ± 0.06
	Probiotic	0.92 ± 0.12	1.23 ± 0.40	0.80 ± 0.05	2.09 ± 0.14
	Synbiotic	0.95 ± 0.13	1.27 ± 0.20	1.16 ± 0.02	1.20 ± 0.24
ICAM	Control	1.24 ± 0.21	2.67 ± 0.18	1.18 ± 0.14	2.35 ± 0.15
	BDPP	2.08 ± 0.20	1.99 ± 0.28	1.04 ± 0.17	0.87 ± 0.06
	Probiotic	1.30 ± 0.20	1.48 ± 0.19	1.06 ± 0.04	1.02 ± 0.47
	Synbiotic	1.40 ± 0.19	1.04 ± 0.15	1.24 ± 0.18	0.47 ± 0.13
VCAM	Control	1.16 ± 0.11	1.98 ± 0.18	0.85 ± 0.15	2.08 ± 0.23
	BDPP	1.02 ± 0.19	1.78 ± 0.19	1.02 ± 0.17	2.01 ± 0.16
	Probiotic	0.60 ± 0.15	2.00 ± 0.17	1.13 ± 0.15	1.47 ± 0.12
	Synbiotic	0.81 ± 0.25	1.74 ± 0.10	1.21 ± 0.11	1.17 ± 0.16

Supplementary Table 5: Ileal Inflammatory Cytokine Expression. Detailed cytokine expression analysis of data presented in figure 4c. Each value represents the fold change of the absolute cytokine expression between the stressed and non-stressed groups. Each value is the mean of n = 6 independent samples +/- SEM.

		US	CUS	CUS+Rest	CUS+US
IL-17A	Control	1.35 ± 0.25	2.15 ± 0.12	0.78 ± 0.10	1.40 ± 0.21
	BDPP	1.08 ± 0.14	1.87 ± 0.21	1.03 ± 0.17	0.62 ± 0.07
	Probiotic	1.19 ± 0.20	1.29 ± 0.09	1.07 ± 0.24	1.00 ± 0.13
	Synbiotic	1.08 ± 0.19	1.07 ± 0.06	0.85 ± 0.20	1.33 ± 0.20
IL-1b	Control	0.95 ± 0.11	1.83 ± 0.22	0.64 ± 0.09	1.26 ± 0.31
	BDPP	0.86 ± 0.12	0.88 ± 0.20	0.60 ± 0.10	0.51 ± 0.07
	Probiotic	0.73 ± 0.21	0.76 ± 0.19	0.52 ± 0.15	1.19 ± 0.21
	Synbiotic	0.72 ± 0.11	0.65 ± 0.12	0.94 ± 0.15	0.82 ± 0.31
IL-6	Control	0.86 ± 0.31	1.99 ± 0.09	0.77 ± 0.13	1.61 ± 0.15
	BDPP	1.75 ± 0.21	0.93 ± 0.07	0.97 ± 0.20	0.76 ± 0.10
	Probiotic	1.21 ± 0.15	1.39 ± 0.22	0.68 ± 0.12	1.03 ± 0.25
	Synbiotic	1.43 ± 0.22	0.85 ± 0.24	0.88 ± 0.12	0.90 ± 0.05
IL-10	Control	1.03 ± 0.11	1.34 ± 0.21	0.76 ± 0.14	0.92 ± 0.14
	BDPP	0.96 ± 0.23	1.62 ± 0.10	0.91 ± 0.10	1.14 ± 0.20
	Probiotic	0.77 ± 0.21	1.83 ± 0.31	0.58 ± 0.08	0.62 ± 0.08
	Synbiotic	1.82 ± 0.13	1.32 ± 0.21	1.06 ± 0.21	1.16 ± 0.09
Kynurenine	Control	0.83 ± 0.12	2.53 ± 0.30	1.04 ± 0.10	2.11 ± 0.21
	BDPP	1.25 ± 0.20	0.87 ± 0.16	0.84 ± 0.17	0.85 ± 0.15
	Probiotic	1.34 ± 0.18	1.36 ± 0.22	0.93 ± 0.20	0.48 ± 0.41
	Synbiotic	1.64 ± 0.21	0.62 ± 0.11	1.05 ± 0.17	0.70 ± 0.20

Supplementary Table 6: Ileal Immune Cell Marker Gene Expression. Detailed gene expression analysis of data presented in figure 4d. Each value is represented as the fold change of the relative gene expression (relative to *gapdh*) from stressed vs. unstressed controls at the same timepoint. Each value is the mean of n = 6 independent samples +/- SEM.

		US	CUS	CUS+Rest	CUS+US
Foxp3	Control	1.20 ± 0.34	1.07 ± 0.21	1.17 ± 0.09	1.58 ± 0.18
	BDPP	1.15 ± 0.17	1.09 ± 0.06	1.57 ± 0.14	1.89 ± 0.27
	Probiotic	1.24 ± 0.18	0.77 ± 0.10	1.23 ± 0.12	1.50 ± 0.21
	Synbiotic	1.69 ± 0.10	1.75 ± 0.19	1.65 ± 0.15	1.95 ± 0.11
Rorc	Control	1.20 ± 0.10	1.76 ± 0.14	0.98 ± 0.20	2.49 ± 0.24
	BDPP	1.69 ± 0.12	1.42 ± 0.18	0.84 ± 0.19	2.98 ± 0.10
	Probiotic	1.15 ± 0.09	1.63 ± 0.19	1.13 ± 0.13	1.92 ± 0.16
	Synbiotic	1.70 ± 0.11	1.69 ± 0.20	1.18 ± 0.13	2.37 ± 0.18
Gata3	Control	0.84 ± 0.42	1.02 ± 0.21	1.46 ± 0.15	1.08 ± 0.11
	BDPP	0.99 ± 0.11	1.02 ± 0.16	1.92 ± 0.17	0.89 ± 0.25
	Probiotic	0.98 ± 0.31	0.90 ± 0.17	1.29 ± 0.17	1.50 ± 0.20
	Synbiotic	1.32 ± 0.17	1.12 ± 0.19	2.03 ± 0.17	1.68 ± 0.21
Tbet	Control	1.33 ± 0.13	1.13 ± 0.14	1.02 ± 0.26	0.76 ± 0.17
	BDPP	0.82 ± 0.42	1.33 ± 0.15	1.48 ± 0.42	1.73 ± 0.31
	Probiotic	1.07 ± 0.19	0.75 ± 0.27	0.72 ± 0.18	1.12 ± 0.11
	Synbiotic	1.13 ± 0.14	1.18 ± 0.29	1.05 ± 0.25	1.08 ± 0.13
Ahr	Control	1.22 ± 0.13	1.00 ± 0.13	1.30 ± 0.06	1.14 ± 0.13
	BDPP	1.26 ± 0.13	1.19 ± 0.13	1.36 ± 0.16	1.72 ± 0.10
	Probiotic	1.12 ± 0.14	1.00 ± 0.14	1.27 ± 0.12	1.43 ± 0.14
	Synbiotic	1.42 ± 0.14	1.31 ± 0.25	1.26 ± 0.21	1.31 ± 0.10
Cyp1a1	Control	0.72 ± 0.08	2.24 ± 0.16	0.86 ± 0.12	2.59 ± 0.21
	BDPP	0.40 ± 0.28	2.27 ± 0.31	1.11 ± 0.18	2.46 ± 0.25
	Probiotic	0.64 ± 0.31	2.55 ± 0.18	0.80 ± 0.03	2.48 ± 0.18
	Synbiotic	1.02 ± 0.17	1.27 ± 0.15	0.74 ± 0.11	1.64 ± 0.15

Supplementary Table 7: Prefrontal Cortex Inflammatory Cytokine Expression. Detailed cytokine expression analysis of data presented in Supplementary Figure 2. Each value represents the fold change of the absolute cytokine expression between the stressed and non-stressed groups. Each value is the mean of n = 6 independent samples +/- SEM.

		US	CUS	CUS+Rest	CUS+US
IL-1b	Control	0.99 ± 0.17	1.71 ± 0.13	1.10 ± 0.10	0.77 ± 0.06
	BDPP	1.41 ± 0.22	1.22 ± 0.20	1.02 ± 0.19	0.96 ± 0.11
	Probiotic	1.44 ± 0.20	1.44 ± 0.11	1.05 ± 0.22	1.00 ± 0.31
	Synbiotic	1.32 ± 0.09	0.83 ± 0.08	0.91 ± 0.07	1.06 ± 0.22
IL-6	Control	1.05 ± 0.13	0.87 ± 0.11	0.97 ± 0.21	1.00 ± 0.21
	BDPP	1.12 ± 0.22	0.98 ± 0.21	1.04 ± 0.25	0.93 ± 0.13
	Probiotic	0.98 ± 0.06	1.05 ± 0.30	0.99 ± 0.29	0.89 ± 0.13
	Synbiotic	0.99 ± 0.15	1.09 ± 0.18	1.00 ± 0.11	0.85 ± 0.14
IL-10	Control	0.90 ± 0.10	1.17 ± 0.13	1.18 ± 0.29	1.10 ± 0.07
	BDPP	0.83 ± 0.03	1.33 ± 0.20	1.04 ± 0.20	1.32 ± 0.14
	Probiotic	1.29 ± 0.10	1.78 ± 0.21	1.17 ± 0.10	1.14 ± 0.11
	Synbiotic	1.47 ± 0.07	1.15 ± 0.22	0.78 ± 0.13	1.00 ± 0.10

Supplementary Table 8: Hippocampus Inflammatory Cytokine Expression. Detailed cytokine expression analysis of data presented in Supplementary Figure 2. Each value represents the fold change of the absolute cytokine expression between the stressed and non-stressed groups. Each value is the mean of n = 6 independent samples +/- SEM.

		US	CUS	CUS+Rest	CUS+US
IL-1b	Control	1.50 ± 0.21	1.18 ± 0.09	0.80 ± 0.27	0.80 ± 0.22
	BDPP	1.35 ± 0.11	0.63 ± 0.22	0.97 ± 0.18	1.06 ± 0.14
	Probiotic	0.88 ± 0.10	0.92 ± 0.21	0.53 ± 0.05	1.04 ± 0.35
	Synbiotic	0.93 ± 0.22	0.72 ± 0.10	1.03 ± 0.21	1.05 ± 0.29
IL-6	Control	1.30 ± 0.24	1.03 ± 0.71	1.13 ± 0.14	0.99 ± 0.28
	BDPP	1.07 ± 0.14	0.80 ± 0.19	1.18 ± 0.14	1.18 ± 0.16
	Probiotic	1.12 ± 0.21	0.80 ± 0.23	1.07 ± 0.12	1.12 ± 0.18
	Synbiotic	0.96 ± 0.22	0.72 ± 0.13	0.89 ± 0.11	0.86 ± 0.11
IL-10	Control	1.08 ± 0.34	0.99 ± 0.34	0.78 ± 0.17	0.96 ± 0.14
	BDPP	0.88 ± 0.12	0.78 ± 0.21	1.23 ± 0.72	1.62 ± 0.32
	Probiotic	1.25 ± 0.14	1.10 ± 0.22	0.95 ± 0.17	0.75 ± 0.18
	Synbiotic	1.22 ± 0.21	0.93 ± 0.19	0.70 ± 0.12	0.97 ± 0.21

Supplementary Table 9: Hippocampal Immune Cell Marker Gene Expression. Detailed gene expression analysis of data presented in Supplementary Figure 2. Each value is represented as the fold change of the relative gene expression (relative to *gapdh*) from stressed vs. unstressed controls at the same timepoint. Each value is the mean of n = 6 independent samples +/- SEM.

		US	CUS	CUS+Rest	CUS+US
Tlr4	Control	0.76 ± 0.12	1.93 ± 0.13	1.14 ± 0.15	2.10 ± 0.14
	BDPP	0.77 ± 0.15	2.37 ± 0.33	0.96 ± 0.24	2.34 ± 0.12
	Probiotic	0.80 ± 0.21	2.76 ± 0.27	1.05 ± 0.16	2.22 ± 0.22
	Synbiotic	0.90 ± 0.12	1.99 ± 0.16	1.79 ± 0.13	2.55 ± 0.12
Nfkb	Control	0.82 ± 0.13	1.46 ± 0.22	1.23 ± 0.23	1.46 ± 0.19
	BDPP	0.95 ± 0.13	1.15 ± 0.29	1.52 ± 0.17	1.99 ± 0.17
	Probiotic	0.83 ± 0.17	2.06 ± 0.25	1.13 ± 0.26	1.51 ± 0.25
	Synbiotic	1.03 ± 0.11	1.29 ± 0.31	1.39 ± 0.21	1.70 ± 0.14
Casp1	Control	1.03 ± 0.16	1.00 ± 0.35	1.43 ± 0.28	0.75 ± 0.15
	BDPP	0.63 ± 0.41	1.58 ± 0.42	1.06 ± 0.21	1.57 ± 0.30
	Probiotic	1.11 ± 0.19	2.69 ± 0.25	0.95 ± 0.18	1.08 ± 0.30
	Synbiotic	0.64 ± 0.12	1.42 ± 0.14	1.28 ± 0.16	0.92 ± 0.17
Il1b	Control	1.10 ± 0.31	1.24 ± 0.13	1.09 ± 0.26	1.75 ± 0.18
	BDPP	0.95 ± 0.16	1.33 ± 0.19	0.81 ± 0.63	1.05 ± 0.30
	Probiotic	1.41 ± 0.21	2.84 ± 0.15	0.60 ± 0.18	1.38 ± 0.13
	Synbiotic	1.59 ± 0.28	3.13 ± 0.30	1.53 ± 0.41	1.02 ± 0.17
Ccl2	Control	0.70 ± 0.12	0.99 ± 0.17	0.74 ± 0.23	0.52 ± 0.16
	BDPP	0.52 ± 0.15	1.00 ± 0.25	0.41 ± 0.29	0.54 ± 0.11
	Probiotic	0.44 ± 0.12	1.52 ± 0.27	0.81 ± 0.16	0.77 ± 0.12
	Synbiotic	1.47 ± 0.42	0.73 ± 0.25	1.34 ± 0.14	1.32 ± 0.29
Ccl5	Control	1.90 ± 0.52	1.46 ± 0.17	1.00 ± 0.13	1.71 ± 0.26
	BDPP	0.71 ± 0.12	1.33 ± 0.15	1.02 ± 0.15	1.18 ± 0.31
	Probiotic	0.84 ± 0.13	1.70 ± 0.25	1.26 ± 0.27	0.90 ± 0.31
	Synbiotic	1.0 ± 0.19	0.87 ± 0.17	1.37 ± 0.22	0.84 ± 0.21
Icam	Control	0.78 ± 0.14	1.01 ± 0.81	1.04 ± 0.41	0.98 ± 0.22
	BDPP	0.66 ± 0.26	0.90 ± 0.16	1.23 ± 0.29	0.29 ± 0.45
	Probiotic	0.95 ± 0.31	1.80 ± 0.13	0.72 ± 0.14	0.41 ± 0.23
	Synbiotic	1.63 ± 0.17	1.80 ± 0.23	1.18 ± 0.31	0.31 ± 0.31
Vcam	Control	0.86 ± 0.18	0.85 ± 0.31	0.89 ± 0.25	1.09 ± 0.16
	BDPP	0.70 ± 0.18	0.73 ± 0.27	0.93 ± 0.28	0.53 ± 0.42
	Probiotic	0.72 ± 0.22	1.18 ± 0.14	0.79 ± 0.30	1.10 ± 0.43
	Synbiotic	1.09 ± 0.32	0.80 ± 0.20	1.07 ± 0.28	0.98 ± 0.26

Supplementary Table 10: Cortex Inflammatory Cytokine Expression. Detailed cytokine expression analysis of data presented in Supplementary Figure 2. Each value represents the fold change of the absolute cytokine expression between the stressed and non-stressed groups. Each value is the mean of n = 6 independent samples +/- SEM.

		US	CUS	CUS+Rest	CUS+US
IL-1b	Control	1.34 ± 0.14	1.54 ± 0.18	0.57 ± 0.34	0.94 ± 0.32
	BDPP	1.22 ± 0.19	1.70 ± 0,24	0.80 ± 0.26	1.04 ± 0.31
	Probiotic	0.85 ± 0.17	0.95 ± 0.22	0.78 ± 0.21	0.77 ± 0.21
	Synbiotic	1.05 ± 0.15	1.10 ± 0.10	0.93 ± 0.32	1.56 ± 0.31
IL-6	Control	1.08 ± 0.08	0.83 ± 0.09	0.86 ± 0.16	1.21 ± 0.16
	BDPP	1.05 ± 0.21	0.43 ± 0.12	1.88 ± 0.23	0.92 ± 0.21
	Probiotic	1.01 ± 0.11	1.04 ± 0.25	1.48 ± 0.31	0.38 ± 0.30
	Synbiotic	1.01 ± 0.21	0.68 ± 0.18	1.50 ± 0.21	0.94 ± 0.32
IL-10	Control	1.21 ± 0.21	1.63 ± 0.13	0.63 ± 0.12	0.97 ± 0.17
	BDPP	1.25 ± 0.31	1.40 ± 0.21	0.85 ± 0.21	0.88 ± 0.21
	Probiotic	1.10 ± 0.17	0.89 ± 0.19	0.72 ± 0.18	0.97 ± 0.16
	Synbiotic	1.37 ± 0.21	0.90 ± 0.17	1.00 ± 0.13	1.07 ± 0.22

Supplementary Table 11: Cortex Immune Cell Marker Gene Expression. Detailed gene expression analysis of data presented in Supplementary Figure 2. Each value is represented as the fold change of the relative gene expression (relative to *gapdh*) from stressed vs. unstressed controls at the same timepoint. Each value is the mean of n = 6 independent samples +/- SEM.

		US	CUS	CUS+Rest	CUS+US
Tlr4	Control	0.91 ± 0.16	1.41 ± 0.19	1.48 ± 0.15	1.26 ± 0.18
	BDPP	0.97 ± 0.06	1.47 ± 0.27	1.32 ± 0.13	1.26 ± 0.17
	Probiotic	0.85 ± 0.20	1.41 ± 0.17	1.07 ± 0.08	1.04 ± 0.19
	Synbiotic	1.02 ± 0.16	1.42 ± 0.17	1.33 ± 0.07	0.84 ± 0.18
Nfkb	Control	0.85 ± 0.09	0.92 ± 0.14	0.88 ± 0.17	1.23 ± 0.11
	BDPP	0.84 ± 0.11	0.82 ± 0.27	0.86 ± 0.26	1.29 ± 0.20
	Probiotic	0.95 ± 0.20	1.30 ± 0.31	0.58 ± 0.45	0.96 ± 0.11
	Synbiotic	0.97 ± 0.20	0.80 ± 0.15	1.02 ± 0.15	0.99 ± 0.16
Casp1	Control	1.58 ± 0.24	1.40 ± 0.23	0.50 ± 0.09	1.05 ± 0.15
	BDPP	1.71 ± 0.25	1.74 ± 0.24	0.66 ± 0.05	1.20 ± 0.25
	Probiotic	1.38 ± 0.36	1.24 ± 0.22	0.60 ± 0.16	1.07 ± 0.20
	Synbiotic	1.06 ± 0.28	1.03 ± 0.22	0.76 ± 0.19	1.63 ± 0.24
Il1b	Control	1.06 ± 0.13	2.62 ± 0.30	1.50 ± 0.24	1.71 ± 0.25
	BDPP	0.73 ± 0.16	1.76 ± 0.16	1.08 ± 0.34	0.90 ± 0.17
	Probiotic	1.12 ± 0.16	2.66 ± 0.31	1.50 ± 0.19	0.84 ± 0.14
	Synbiotic	1.06 ± 0.18	2.68 ± 0.30	0.93 ± 0.17	1.43 ± 0.30
Ccl2	Control	1.13 ± 0.34	1.04 ± 0.19	0.61 ± 0.19	0.95 ± 0.23
	BDPP	0.76 ± 0.29	1.30 ± 0.53	0.79 ± 0.20	0.68 ± 0.14
	Probiotic	1.95 ± 0.26	2.11 ± 0.22	0.41 ± 0.32	1.14 ± 0.11
	Synbiotic	1.56 ± 0.22	1.98 ± 0.16	0.30 ± 0.12	1.46 ± 0.16
Ccl5	Control	1.16 ± 0.19	1.72 ± 0.22	0.70 ± 0.30	1.00 ± 0.14
	BDPP	0.72 ± 0.30	1.32 ± 0.29	0.61 ± 0.13	1.31 ± 0.16
	Probiotic	0.77 ± 0.18	1.50 ± 0.26	1.01 ± 0.17	0.90 ± 0.30
	Synbiotic	1.11 ± 0.16	1.43 ± 0.28	0.51 ± 0.14	1.05 ± 0.18
Icam	Control	0.99 ± 0.06	2.33 ± 0.18	2.47 ± 0.40	1.89 ± 0.17
	BDPP	0.60 ± 0.22	1.49 ± 0.15	1.98 ± 0.38	2.62 ± 0.25
	Probiotic	0.87 ± 0.15	3.60 ± 0.17	1.29 ± 0.34	2.18 ± 0.31
	Synbiotic	1.68 ± 0.21	1.48 ± 0.22	0.54 ± 0.14	1.58 ± 0.44
Vcam	Control	0.92 ± 0.22	0.98 ± 0.25	1.19 ± 0.14	1.17 ± 0.15
	BDPP	0.83 ± 0.10	1.03 ± 0.28	1.16 ± 0.10	1.22 ± 0.25
	Probiotic	0.83 ± 0.02	1.55 ± 0.13	0.69 ± 0.19	1.50 ± 0.31
	Synbiotic	0.97 ± 0.09	1.83 ± 0.24	0.67 ± 0.25	1.06 ± 0.21

Supplementary Table 12: Cerebellum Inflammatory Cytokine Expression. Detailed cytokine expression analysis of data presented in Supplementary Figure 2. Each value represents the fold change of the absolute cytokine expression between the stressed and non-stressed groups. Each value is the mean of n = 6 independent samples +/- SEM.

		US	CUS	CUS+Rest	CUS+US
IL-1b	Control	0.92 ± 0.08	0.95 ± 0.06	0.93 ± 0.13	0.76 ± 0.14
	BDPP	1.19 ± 0.09	1.02 ± 0.21	1.23 ± 0.15	0.69 ± 0.21
	Probiotic	1.07 ± 0.22	0.76 ± 0.15	0.64 ± 0.21	0.82 ± 0.12
	Synbiotic	1.39 ± 0.21	0.78 ± 0.21	0.89 ± 0.22	0.88 ± 0.21
IL-6	Control	0.68 ± 0.21	0.86 ± 0.14	0.83 ± 0.13	0.63 ± 0.04
	BDPP	0.78 ± 0.15	0.86 ± 0.21	1.36 ± 0.41	0.72 ± 0.09
	Probiotic	1.30 ± 0.22	0.96 ± 0.22	0.88 ± 0.15	0.97 ± 0.20
	Synbiotic	1.45 ± 0.21	0.84 ± 0.29	1.00 ± 0.25	1.11 ± 0.25
IL-10	Control	0.80 ± 0.28	1.58 ± 0.21	0.73 ± 0.14	1.01 ± 0.21
	BDPP	0.83 ± 0.19	1.30 ± 0.19	1.03 ± 0.25	0.93 ± 0.14
	Probiotic	0.95 ± 0.21	0.90 ± 0.17	0.70 ± 0.21	0.87 ± 0.21
	Synbiotic	0.86 ± 0.15	1.25 ± 0.21	0.93 ± 0.22	2.41 ± 0.31

Supplementary Table 13: Cortex Immune Cell Marker Gene Expression. Detailed gene expression analysis of data presented in Supplementary Figure 2. Each value is represented as the fold change of the relative gene expression (relative to *gapdh*) from stressed vs. unstressed controls at the same timepoint. Each value is the mean of n = 6 independent samples +/- SEM.

		US	CUS	CUS+Rest	CUS+US
Tlr4	Control	0.99 ± 0.17	0.85 ± 0.16	1.04 ± 0.19	0.95 ± 0.15
	BDPP	0.79 ± 0.26	0.82 ± 0.17	1.11 ± 0.23	1.07 ± 0.29
	Probiotic	1.46 ± 0.20	0.84 ± 0.13	0.98 ± 0.29	0.81 ± 0.19
	Synbiotic	1.26 ± 0.20	1.63 ± 0.17	0.84 ± 0.27	1.02 ± 0.17
Nfkb	Control	0.87 ± 0.15	1.31 ± 0.24	1.37 ± 0.17	1.64 ± 0.18
	BDPP	1.02 ± 0.61	1.02 ± 0.15	0.83 ± 0.19	1.63 ± 0.14
	Probiotic	1.05 ± 0.21	0.70 ± 0.16	0.64 ± 0.32	1.34 ± 0.17
	Synbiotic	1.25 ± 0.31	0.75 ± 0.19	0.95 ± 0.21	3.70 ± 0.34
Casp1	Control	1.17 ± 0.21	1.30 ± 0.25	1.18 ± 0.13	1.05 ± 0.21
	BDPP	0.64 ± 0.12	1.21 ± 0.10	1.38 ± 0.19	0.74 ± 0.19
	Probiotic	1.38 ± 0.10	1.32 ± 0.09	1.40 ± 0.22	1.06 ± 0.14
	Synbiotic	1.84 ± 0.16	1.05 ± 0.24	1.39 ± 0.16	0.96 ± 0.21
Il1b	Control	0.92 ± 0.22	2.63 ± 0.40	2.32 ± 0.13	1.67 ± 0.16
	BDPP	0.28 ± 0.59	1.06 ± 0.32	0.82 ± 0.16	1.14 ± 0.17
	Probiotic	1.25 ± 0.43	1.02 ± 0.27	0.66 ± 0.12	1.29 ± 0.15
	Synbiotic	0.86 ± 0.19	0.37 ± 0.30	0.57 ± 0.15	2.49 ± 0.19
Ccl2	Control	0.75 ± 0.20	1.81 ± 0.12	0.91 ± 0.21	0.93 ± 0.20
	BDPP	0.79 ± 0.27	1.16 ± 0.22	0.80 ± 0.15	1.16 ± 0.13
	Probiotic	1.26 ± 0.20	1.02 ± 0.22	1.01 ± 0.20	1.39 ± 0.23
	Synbiotic	1.03 ± 0.12	1.22 ± 0.14	1.18 ± 0.17	1.01 ± 0.17
Ccl5	Control	0.85 ± 0.29	2.07 ± 0.24	1.13 ± 0.13	1.53 ± 0.15
	BDPP	0.98 ± 0.23	1.24 ± 0.17	1.10 ± 0.21	1.48 ± 0.22
	Probiotic	0.72 ± 0.42	1.66 ± 0.18	0.91 ± 0.11	1.18 ± 0.21
	Synbiotic	0.78 ± 0.17	1.09 ± 0.12	1.29 ± 0.14	1.51 ± 0.17
Icam	Control	1.28 ± 0.15	1.37 ± 0.26	2.11 ± 0.16	2.67 ± 0.19
	BDPP	0.57 ± 0.18	0.60 ± 0.07	0.72 ± 0.09	1.39 ± 0.12
	Probiotic	2.16 ± 0.18	0.81 ± 0.20	2.02 ± 0.16	1.45 ± 0.15
	Synbiotic	1.60 ± 0.14	0.55 ± 0.06	1.80 ± 0.19	1.14 ± 0.24
Vcam	Control	1.13 ± 0.20	0.90 ± 0.14	0.89 ± 0.14	1.38 ± 0.14
	BDPP	0.92 ± 0.16	0.85 ± 0.15	0.54 ± 0.22	0.76 ± 0.12
	Probiotic	1.71 ± 0.23	0.66 ± 0.13	0.96 ± 0.20	1.07 ± 0.21
	Synbiotic	1.67 ± 0.27	0.62 ± 0.18	1.00 ± 0.14	1.44 ± 0.18

Supplementary Table 14: Spleen Immune Cell Marker Gene Expression. Detailed gene expression analysis of data presented in Supplementary Figure 8. Each value is represented as the fold change of the relative gene expression (relative to *gapdh*) from stressed vs. unstressed controls at the same timepoint. Each value is the mean of n = 6 independent samples +/- SEM, where the SEM was calculated as the geometric mean of the SEM of the two groups used to build the ratio.

		US	CUS	CUS+Rest	CUS+US
Foxp3	Control	1.25 ± 0.21	0.84 ± 0.12	1.26 ± 0.19	1.21 ± 0.13
	BDPP	1.70 ± 0.30	0.91 ± 0.08	1.32 ± 0.21	1.84 ± 0.13
	Probiotic	1.01 ± 0.12	0.69 ± 0.12	0.91 ± 0.16	1.15 ± 0.13
	Synbiotic	1.22 ± 0.08	0.84 ± 0.24	0.80 ± 0.21	1.94 ± 0.23
Rorc	Control	1.58 ± 0.18	3.49 ± 0.35	1.83 ± 0.16	0.90 ± 0.13
	BDPP	2.01 ± 0.16	3.35 ± 0.53	1.55 ± 0.23	1.83 ± 0.26
	Probiotic	0.84 ± 0.17	2.75 ± 0.39	1.12 ± 0.30	0.81 ± 0.16
	Synbiotic	1.06 ± 0.17	1.96 ± 0.30	1.03 ± 0.16	0.96 ± 0.41
Gata3	Control	1.49 ± 0.10	1.19 ± 0.09	1.14 ± 0.22	0.82 ± 0.30
	BDPP	1.11 ± 0.15	1.16 ± 0.13	1.26 ± 0.21	1.21 ± 0.16
	Probiotic	0.92 ± 0.09	1.24 ± 0.08	1.34 ± 0.35	0.90 ± 0.16
	Synbiotic	1.09 ± 0.06	1.31 ± 0.10	1.16 ± 0.11	1.12 ± 0.18
Tbet	Control	0.76 ± 0.22	0.94 ± 0.17	1.15 ± 0.20	0.86 ± 0.12
	BDPP	1.08 ± 0.20	1.05 ± 0.09	1.94 ± 0.40	1.74 ± 0.17
	Probiotic	0.84 ± 0.19	1.07 ± 0.16	0.95 ± 0.17	0.73 ± 0.18
	Synbiotic	0.72 ± 0.14	0.72 ± 0.13	1.21 ± 0.29	1.11 ± 0.28
Ahr	Control	0.87 ± 0.23	0.87 ± 0.11	1.24 ± 0.22	0.74 ± 0.19
	BDPP	0.83 ± 0.19	0.78 ± 0.16	1.32 ± 0.18	0.81 ± 0.26
	Probiotic	0.85 ± 0.17	0.73 ± 0.15	1.28 ± 0.12	0.83 ± 0.15
	Synbiotic	0.87 ± 0.13	0.72 ± 0.12	0.87 ± 0.22	0.75 ± 0.17
Cyp1a1	Control	1.06 ± 0.23	2.00 ± 0.12	1.21 ± 0.18	2.14 ± 0.21
	BDPP	0.94 ± 0.17	1.07 ± 0.19	0.94 ± 0.17	1.94 ± 0.41
	Probiotic	0.70 ± 0.14	0.86 ± 0.13	1.09 ± 0.19	2.29 ± 0.30
	Synbiotic	1.08 ± 0.19	0.61 ± 0.15	1.26 ± 0.13	1.27 ± 0.21
Tlr4	Control	0.93 ± 0.11	1.20 ± 0.16	0.85 ± 0.20	0.96 ± 0.17
	BDPP	0.97 ± 0.21	1.02 ± 0.14	1.11 ± 0.16	1.13 ± 0.15
	Probiotic	0.86 ± 0.22	1.28 ± 0.20	0.86 ± 0.24	1.08 ± 0.13
	Synbiotic	0.94 ± 0.22	1.02 ± 0.15	0.76 ± 0.25	1.07 ± 0.14
Nfkb	Control	0.85 ± 0.11	0.95 ± 0.12	1.08 ± 0.17	0.88 ± 0.11
	BDPP	0.80 ± 0.10	0.88 ± 0.11	1.23 ± 0.21	1.07 ± 0.21
	Probiotic	0.78 ± 0.16	1.21 ± 0.11	0.89 ± 0.16	0.82 ± 0.18
	Synbiotic	0.78 ± 0.18	1.24 ± 0.20	0.91 ± 0.16	0.91 ± 0.14
Il1b	Control	0.72 ± 0.16	1.23 ± 0.14	1.44 ± 0.23	1.11 ± 0.15
	BDPP	0.77 ± 0.21	0.78 ± 0.19	0.91 ± 0.22	0.74 ± 0.10
	Probiotic	0.74 ± 0.18	1.55 ± 0.19	1.04 ± 0.32	0.92 ± 0.13
	Synbiotic	1.01 ± 0.26	1.47 ± 0.17	1.16 ± 0.31	0.94 ± 0.16
Ido	Control	0.45 ± 0.37	0.80 ± 0.11	0.86 ± 0.18	0.87 ± 0.18
	BDPP	0.97 ± 0.12	1.18 ± 0.21	1.63 ± 0.35	0.56 ± 0.17
	Probiotic	0.71 ± 0.20	0.79 ± 0.15	0.83 ± 0.44	0.51 ± 0.12
	Synbiotic	0.68 ± 0.20	1.02 ± 0.17	0.82 ± 0.87	0.51 ± 0.16

Supplementary Table 15: Splenic Inflammatory Cytokine Expression. Detailed cytokine expression analysis of data presented in Supplementary Figure 8. Each value represents the fold change of the absolute cytokine expression between the stressed and non-stressed groups. Each value is the mean of n = 6 independent samples +/- SEM.

		US	CUS	CUS+Rest	CUS+US
IL-1b	Control	1.02 ± 0.08	1.34 ± 0.15	1.52 ± 0.21	1.00 ± 0.21
	BDPP	1.02 ± 0.11	1.03 ± 0.18	1.00 ± 0.13	0.47 ± 0.17
	Probiotic	0.93 ± 0.12	0.79 ± 0.21	1.19 ± 0.31	0.65 ± 0.13
	Synbiotic	1.20 ± 0.31	0.52 ± 0.17	1.36 ± 0.21	0.65 ± 0.14
IL-6	Control	0.87 ± 0.04	1.54 ± 0.09	1.01 ± 0.21	1.04 ± 0.30
	BDPP	0.90 ± 0.10	0.99 ± 0.12	1.19 ± 0.12	0.91 ± 0.13
	Probiotic	0.90 ± 0.12	0.61 ± 0.10	1.18 ± 0.17	0.90 ± 0.15
	Synbiotic	1.27 ± 0.31	0.70 ± 0.07	0.97 ± 0.20	0.88 ± 0.17
IL-10	Control	0.99 ± 0.12	0.78 ± 0.13	1.22 ± 0.21	0.68 ± 0.13
	BDPP	0.90 ± 0.31	0.89 ± 0.19	1.28 ± 0.26	1.06 ± 0.11
	Probiotic	1.40 ± 0.41	0.74 ± 0.17	0.94 ± 0.17	0.96 ± 0.12
	Synbiotic	1.36 ± 0.21	0.91 ± 0.04	0.98 ± 0.21	1.36 ± 0.40

Supplementary Table 16: Splenic Inflammatory Cytokine Expression. Detailed cytokine expression analysis of data presented in Supplementary Figure 8. Each value represents the fold change of the absolute cytokine expression between the stressed and non-stressed groups. Each value is the mean of n = 6 independent samples +/- SEM.

		US	CUS	CUS+Rest	CUS+US
IL-1b	Control	1.02 ± 0.12	1.34 ± 0.13	1.52 ± 0.22	1.00 ± 0.14
	BDPP	1.02 ± 0.09	1.03 ± 0.14	1.00 ± 0.16	0.47 ± 0.13
	Probiotic	0.93 ± 0.08	0.79 ± 0.19	1.19 ± 0.06	0.65 ± 0.07
	Synbiotic	1.20 ± 0.11	0.52 ± 0.21	1.36 ± 0.26	0.65 ± 0.12
IL-6	Control	0.87 ± 0.23	1.54 ± 0.13	1.01 ± 0.17	1.04 ± 0.21
	BDPP	0.90 ± 0.27	0.99 ± 0.25	1.19 ± 0.22	0.91 ± 0.14
	Probiotic	0.90 ± 0.21	0.61 ± 0.18	1.18 ± 0.27	0.90 ± 0.21
	Synbiotic	1.27 ± 0.27	0.70 ± 0.20	0.97 ± 0.31	0.88 ± 0.17
IL-10	Control	0.99 ± 0.13	0.78 ± 0.14	1.22 ± 0.30	0.68 ± 0.13
	BDPP	0.90 ± 0.17	0.89 ± 0.13	1.18 ± 0.29	1.06 ± 0.14
	Probiotic	1.40 ± 0.21	0.74 ± 0.23	0.94 ± 0.08	0.96 ± 0.16
	Synbiotic	1.36 ± 0.28	0.91 ± 0.22	0.98 ± 0.09	1.36 ± 0.21

Supplementary Table 17: Serum Inflammatory Cytokine Expression. Detailed cytokine expression analysis of data presented in Supplementary Figure 8. Each value represents the fold change of the absolute cytokine expression between the stressed and non-stressed groups. Each value is the mean of n = 6 independent samples +/- SEM.

		US	CUS	CUS+Rest	CUS+US
IL-1b	Control	1.10 ± 0.09	1.13 ± 0.21	1.15 ± 0.21	1.69 ± 0.31
	BDPP	1.06 ± 0.12	1.10 ± 0.08	0.88 ± 0.09	0.74 ± 0.21
	Probiotic	1.12 ± 0.22	1.11 ± 0.11	1.08 ± 1.12	2.18 ± 0.30
	Synbiotic	0.91 ± 0.21	0.91 ± 0.22	1.13 ± 0.12	0.49 ± 0.17
IL-6	Control	1.28 ± 0.21	1.25 ± 0.16	2.60 ± 0.41	2.15 ± 0.21
	BDPP	0.65 ± 0.08	0.68 ± 0.11	1.12 ± 0.22	0.86 ± 0.21
	Probiotic	0.28 ± 0.34	2.23 ± 0.21	2.61 ± 0.18	2.44 ± 31
	Synbiotic	1.07 ± 0.25	0.72 ± 0.18	0.52 ± 0.28	0.50 ± 0.21
IL-10	Control	1.77 ± 0.21	6.04 ± 0.31	4.46 ± 0.21	5.07 ± 0.31
	BDPP	1.88 ± 0.15	3.61 ± 0.22	2.39 ± 0.24	1.51 ± 0.21
	Probiotic	1.32 ± 0.20	2.19 ± 0.28	0.93 ± 0.09	2.67 ± 0.16
	Synbiotic	1.62 ± 0.17	0.92 ± 0.13	0.17 ± 0.22	0.46 ± 0.21
